# Photocaged Histone Deacetylase Inhibitors as Prodrugs in Targeted Cancer Therapy

**DOI:** 10.3390/ph16030356

**Published:** 2023-02-25

**Authors:** Fabian B. Kraft, Maria Hanl, Felix Feller, Linda Schäker-Hübner, Finn K. Hansen

**Affiliations:** Department of Pharmaceutical and Cell Biological Chemistry, Pharmaceutical Institute, University of Bonn, An der Immenburg 4, 53121 Bonn, Germany

**Keywords:** histone deacetylase, prodrug, photo-activatable, epigenetics, cancer

## Abstract

Histone deacetylases (HDACs) play a key role in the control of transcription, cell proliferation, and migration. FDA-approved histone deacetylase inhibitors (HDACi) demonstrate clinical efficacy in the treatment of different T-cell lymphomas and multiple myeloma. However, due to unselective inhibition, they display a wide range of adverse effects. One approach to avoiding off-target effects is the use of prodrugs enabling a controlled release of the inhibitor in the target tissue. Herein, we describe the synthesis and biological evaluation of HDACi prodrugs with photo-cleavable protecting groups masking the zinc-binding group of the established HDACi DDK137 (**I**) and VK1 (**II**). Initial decaging experiments confirmed that the photocaged HDACi **pc-I** could be deprotected to its parent inhibitor **I**. In HDAC inhibition assays, **pc-I** displayed only low inhibitory activity against HDAC1 and HDAC6. After irradiation with light, the inhibitory activity of **pc-I** strongly increased. Subsequent MTT viability assays, whole-cell HDAC inhibition assays, and immunoblot analysis confirmed the inactivity of **pc-I** at the cellular level. Upon irradiation, **pc-I** demonstrated pronounced HDAC inhibitory and antiproliferative activities which were comparable to the parent inhibitor **I**. Additionally, only phototreated **pc-I** was able to induce apoptosis in Annexin V/PI and caspase-Glo 3/7 assays, making **pc-I** a valuable tool for the development of light-activatable HDACi.

## 1. Introduction

**Figure 1:**
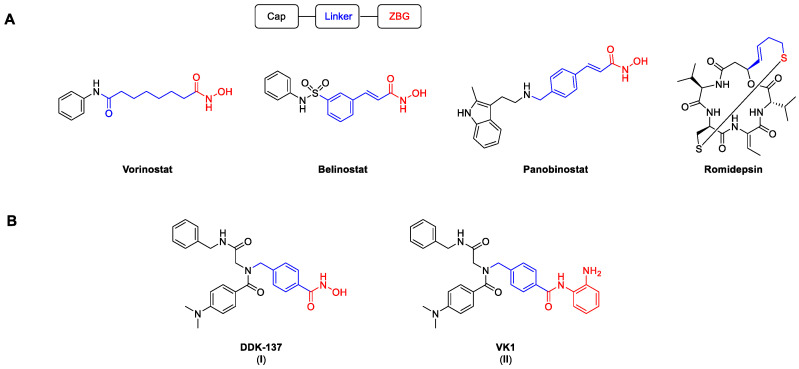
Selected HDACi, including the established HDAC pharmacophore model. Cap, linker, and ZBG are highlighted in black, blue, and red, respectively. (**A**) Structures of FDA-approved HDACi. (**B**) Structures of the peptoid-based HDACi **DDK-137** (**I**) and **VK1** (**II**) [7,8].

Histone deacetylases are a group of enzymes that remove acyl groups from the ε-amino groups of lysine side chains [1]. Despite their name, the target range is not limited only to histone proteins, but also includes numerous non-histone proteins [2,3]. There are 18 known human HDAC isoforms, which can be roughly divided into two families according to their catalytic activities. Eleven isoforms possess a zinc ion in their catalytic domain, while the other seven, the so-called sirtuins, depend on the cofactor NAD^+^. In accordance with their cellular localization and structural homology, the zinc-dependent isoforms are further classified into different groups: class I (HDAC 1, 2, 3, and 8), class IIa (HDAC 4, 5, 7, and 9), class IIb (HDAC 6 and 10), and class IV (HDAC 11) [4]. Through their direct influence on the chromatin structure, HDACs play a significant role in important processes such as transcription, as well as cell proliferation, differentiation, and migration [5]. Their high expression rates in different kinds of cancer have led to the development of numerous HDAC inhibitors (HDACi). As of now, four HDACi are approved by the FDA for the treatment of different T-cell lymphomas and multiple myeloma (Figure 1) [6]. Among them, the pan-HDACi vorinostat, belinostat, and panobinostat possess a hydroxamic acid as a zinc-binding group (ZBG), while the prodrug romidepsin binds via an in situ generated thiol.

Although they are known to be possibly mutagenic, hydroxamic acids are among the most common ZBGs. Notably, vorinostat, panobinostat, and belinostat all show mutagenic activity in the Ames test and provoke chromosomal aberrations in rodent cells [9]. Another considerable issue with hydroxamic-acid-based HDACi is their relatively low metabolic stability in vivo [10]. In addition, all FDA-approved HDACi can cause severe hematological and gastrointestinal side effects, which are presumably caused by non-specific HDAC inhibition and frequently lead to treatment interruptions [11,12,13]. Therefore, the clinical use of unselective HDACi has so far been limited to a small number of cancer entities. 

One approach to circumvent unwanted off-target effects while preserving the therapeutic efficacy is to release or activate the active drug in the target tissue [14]. Photodynamic therapy (PDT) is a classic example for the activation of a drug in the diseased tissue. The PDT principle relies on a photosensitizer, which, after exposure to light of a specific wavelength, produces radicals and/or reactive oxygen species [15,16]. Since the photosensitizer is only activated locally, most of the side effects can be avoided. In addition to the treatment of acne [17], PDT has shown increasing influence on cancer therapy [18,19]. In another approach to avoid unwanted side effects, the active drug can be converted into a prodrug and masked by a photocleavable (pc) protecting group, which allows the release of the parent drug in the target tissue with high tempo-spatial accuracy [14]. In 1977, *Engels* et al. described the photorelease of cAMP, the first biological application of a photoremovable protecting group (PPG) [20]. Today, *o*-nitrobenzyl derivatives are by far the most commonly used PPGs [21]. The main requirement for the application of light-induced treatments is the accessibility of the target tissue to the light source. Consequently, skin cancers are particularly suitable for photopharmacological approaches. Current modern treatments of malignant melanoma are based on BRAF inhibitors like vemurafenib [22] but are accompanied by severe side effects and fast development of resistance. *Pfeifer* and co-workers established a vemurafenib derivative with a PPG [23]. In vitro investigations showed the inactivity of the protected inhibitor, while irradiation with light restored the activity against kinases [23]. Additional recent examples of photocaged drugs in the field of cancer include phosphoinositide 3-kinase (PI3K) [24], mouse double minute 2 homolog (MDM2) [25], and proteasome inhibitors [26]. Furthermore, PPGs have been successfully utilized to transform proteolysis-targeting chimeras (PROTACs) into photo-activatable degraders of the bromodomain-containing protein 4 (BRD4) [27] and estrogen-related receptor α (ERRα) [28].

Eventually, it was discovered that drug-resistant malignant melanoma cells overexpress HDAC1-3, and the inhibition of these isoforms overcomes drug resistance via different mechanisms [29,30]. To minimize off-target effects, the photo-controlled release of HDACi in the target tissue is desirable. In 2016, *Gasser* and co-workers converted the ferrocene-containing HDACi Fc-SAHA into the photocaged analog p-Fc-SAHA. However, the insufficient physicochemical properties of p-Fc-SAHA prohibited the investigation of its properties in a cellular setting [31]. Recently, *Conway* and coworkers developed the panobinostat prodrug Zap-Pano utilizing a 4,5-dimethoxy-2-nitrobenzyl (DMNB) group attached to the hydroxamic acid oxygen. Through irradiation with light at 405 nm, they were able to release the parent inhibitor panobinostat under controlled conditions [32]. While they could completely suppress the HDAC inhibitory activity with their prodrug, another major side product, the corresponding amide Pano-NH_2_, was released upon irradiation with light [32].

We previously reported the synthesis and biological evaluation of the highly potent pan-inhibitor **DDK-137** (**I**) [8,33] and the HDAC1-3 selective inhibitor **VK1** (**II**) [7]. Compound **I** displayed potent antiplasmodial activity against asexual blood stage and liver stage parasites and also submicromolar antiproliferative activity against the HepG2 cancer cell line [8]. The *ortho*-aminoanilide **II** revealed promising antiproliferative effects in the human ovarian cancer cell line A2780 and the human squamous carcinoma cell line Cal27. In addition, **II** demonstrated chemosensitizing properties and was capable of reversing cisplatin resistance in cisplatin-resistant Cal27CisR cells [7]. In this work, we describe the synthesis and biological evaluation of photocaged HDACi prodrugs for both inhibitors.

## 2. Results

### 2.1. Design and Synthesis of HDACi Prodrugs

**Figure 2:**
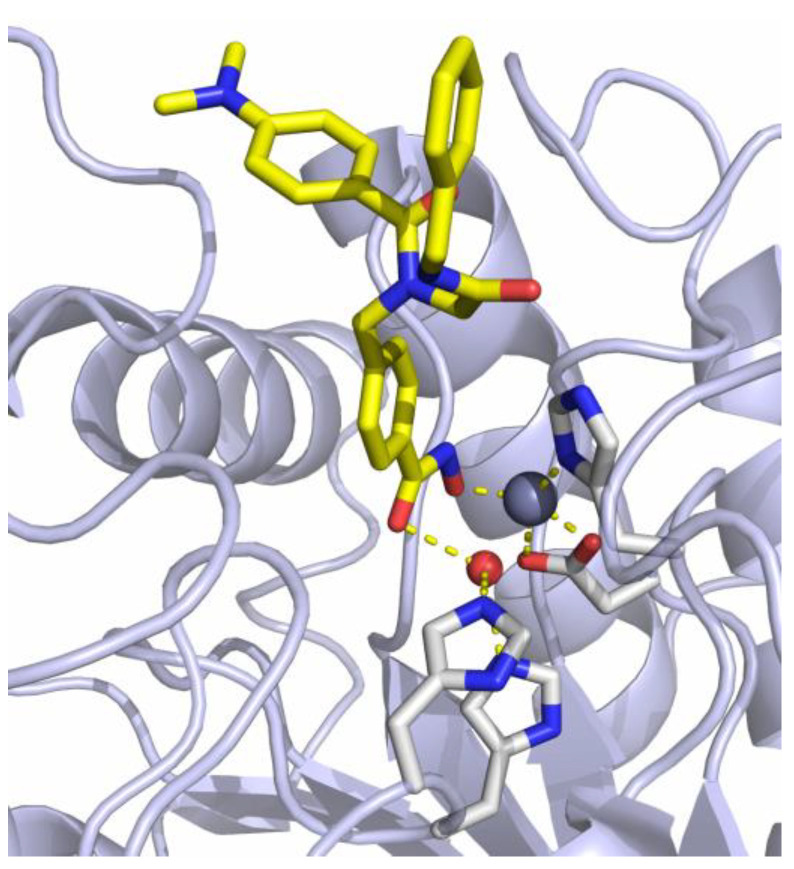
Crystal structure of **I** in complex with HDAC6 (PDB ID: 6DVN). The catalytic Zn^2+^-ion appears as a gray, the water molecule as a red sphere. Metal coordination and hydrogen bond interactions are shown as dashed yellow lines [33].

The previously reported crystal structure of the non-selective HDACi **I** in complex with the second catalytic domain of zebrafish HDAC6 is depicted in Figure 2 [33]. The hydroxamic acid moiety coordinates to the Zn^2+^-ion and the adjacent water molecule at the same time in a monodentate binding mode. Consequently, the inhibitory potential of **I** against Zn^2+^-dependent HDAC enzymes should be diminished by masking the hydroxamic acid warhead.

**Scheme 1:**
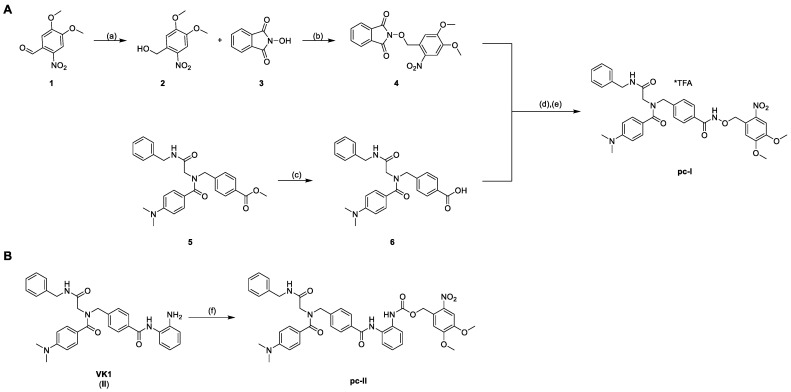
Synthesis of the photocaged prodrugs **pc-I** (**A**) and **pc-II** (**B**). Reagents and conditions: (**a**) NaBH_4_, CH_2_Cl_2_, rt, 30 min, 98%; (**b**) PPh_3_, DIAD, THF, 0 °C to rt, 4 h, quant.; (**c**) 1.25 m NaOH_aq_, THF/MeOH 10/1 *v*/*v*, 40 °C, 24 h, 97%; (**d**) NH_2_NH_2_*H_2_O, CH_2_Cl_2_, rt, 4 h; (**e**) EDC*HCl, CH_2_Cl_2_, 0 °C to rt, 2 h, 16%; (**f**) NVOC-Cl, pyridine, 0 °C to rt, THF, 19 h, 51%.

To enable the optochemical control of HDAC inhibition with a high spatio-temporal accuracy, we attached a photocleavable DMNB moiety to the zinc-binding oxygen atom of the inhibitor. Additionally, we protected the previously reported *ortho*-aminoanilide **II** via a carbamate moiety with a DMNB group (Scheme 1) [7]. The syntheses of the parent inhibitors **I** and **II** were accomplished as previously published [7,8].

The synthesis of the photocaged prodrugs **pc-I** and **pc-II** is summarized in Scheme 1. The *N*-phthalimide protected compound **4** was synthesized via a two-step protocol starting from commercially available 6-nitroverartraldehyde **1**. The reduction of **1** to the corresponding benzyl alcohol **2** was conducted in an excellent yield of 98%. Further on, the *Mitsunobu* reaction of **2** with *N*-hydroxyphthalimide **3** yielded the protected hydroxylamine **4** under mild conditions as a yellow solid. The HDACi precursor **6** was obtained in two steps according to our published protocol. Briefly, an *Ugi* four-component reaction afforded the intermediate **5**, which was hydrolyzed to generate **6**. The subsequent deprotection of **4** with hydrazine followed by an amide coupling with **6** provided the prodrug **pc-I** in a yield of 16% (Scheme 1A). Finally, the photocaged **VK1**-prodrug **pc-II** was prepared in 51% yield via the treatment of **II** with 6-nitroveratryl chloroformate (NVOC-Cl) in the presence of pyridine as a base (Scheme 1B).

### 2.2. Biological Evaluation of HDACi Prodrugs

Subsequently, the photo-uncaging of the two photocaged HDACi was investigated with high-performance liquid chromatography (HPLC) analysis. The photocleavage reaction of **pc-I** in DMSO after irradiation with light at a wavelength of 365 nm proceeded smoothly, and the HPLC chromatograms clearly showed the appearance of compound **I**. After treatment for 10 min, **pc-I** was deprotected to its parent inhibitor **I** in a yield of 82%, as determined by HPLC using a calibration curve. Compound **pc-II** decomposed under treatment with light and did not release the parent HDACi **II** in significant quantities (see Supporting Information). Thus, we focused our biological evaluation on the prodrug **pc-I**. 

In the first step, **pc-I** was screened in fluorogenic assays for its in vitro inhibitory activity against HDAC1 (class I) and HDAC6 (class IIb) with or without UV irradiation (365 nm) (Table 1).

**Figure 3:**
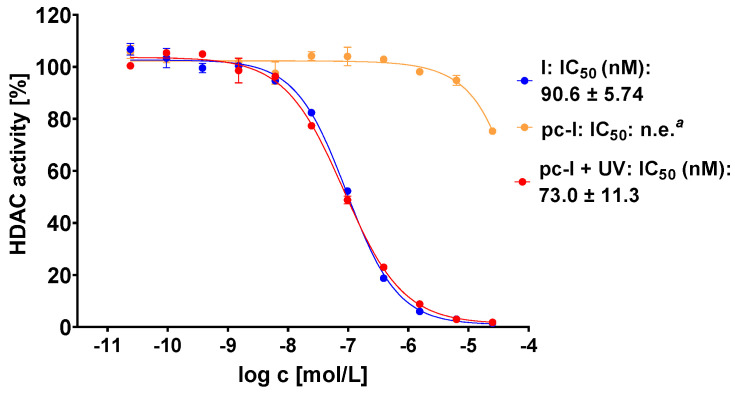
Representative concentration-effect curves of the whole-cell HDAC inhibition assays of **pc-I** and **I** in MV-3 cells. Stated IC_50_ values were determined from the mean of three independent experiments in duplicates. Irradiation with UV light was executed at 365 nm for 10 min. *^a^* n.e: no effect: <25% inhibition at 25 µM.

The photocaged prodrug pc-I showed low inhibitory activity against both isoforms. Upon irradiation with UV-light, we detected considerably increased inhibition of HDAC1 (121-fold) and HDAC6 (37-fold). Encouraged by these enzymatic data, we assessed our prodrug in a whole-cell HDAC assay using the melanoma cell line MV-3 (Figure 3). This cell line was selected because skin cancers are particularly suitable for the light-triggered release of photocaged drugs. Without UV irradiation, **pc-I** showed less than 25% inhibition at 25 µM. After irradiation for 10 min, **pc-I** (IC_50_: 73 nM) demonstrated potent activity in the whole-cell HDAC inhibition assay, which was comparable to the parent compound **I** (IC_50_: 91 nM).

**Figure 4:**
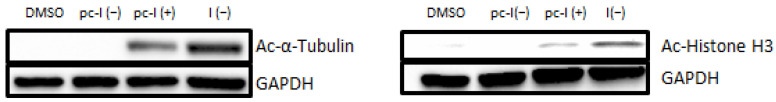
Representative immunoblot analysis of acetylated α-tubulin and histone H3. MV-3 cells were incubated for 24 h with **I** and **pc-I** at a concentration of 3.1 µM. Afterward, cell lysates were immunoblotted with an anti-acetyl-α-tubulin and anti-acetyl-histone H3 antibody. GAPDH was used as loading control. (+): UV irradiation at 365 nm for 10 min. (−): no treatment with UV light.

Next, we performed additional cellular target engagement assessment with immunoblot analysis with the MV-3 cell line (Figure 4). We chose acetyl-α-tubulin (a marker of HDAC6 inhibition) and acetyl-histone H3 (a marker of HDAC1-3 inhibition) as representative HDAC substrates. For **pc-I** without irradiation, no increased levels of acetylated α-tubulin were observed. The parent inhibitor **I** showed strong hyperacetylation, thus proving the inhibition of HDAC6. Similarly, activation of **pc-I** with UV light led to an increased level of acetyl-α-tubulin. The same tendency was observed for the acetylation levels of histone H3. After UV activation of **pc-I**, a higher amount of acetyl-histone-H3 was measured, confirming the inhibition of class I HDAC enzymes in a cellular environment.

**Figure 5:**
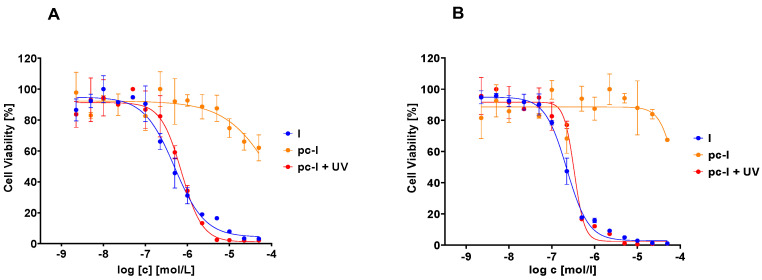
Representative concentration-effect curves of the MTT cell viability assays of **pc-I** and **I**. (**A**) MV-3 cell line, (**B**) A2780 cell line. UV irradiation was executed at 365 nm for 10 min.

Subsequently, we evaluated our compound with in vitro MTT cell viability assays against the MV-3 cell line and the ovarian cancer cell line A2780 (Figure 5, Table 2). In accordance with our previous data, **pc-I** showed <25% inhibition at 25 µM in both cell lines. As expected, upon light activation, pronounced antiproliferative activity was measured (MV-3 IC_50_: 727 nM; A2780 IC_50_: 428 nM). Thus, we confirmed the inactivity of our photocaged inhibitor in a cellular environment and the cell toxicity upon irradiation with light.

**Figure 6:**
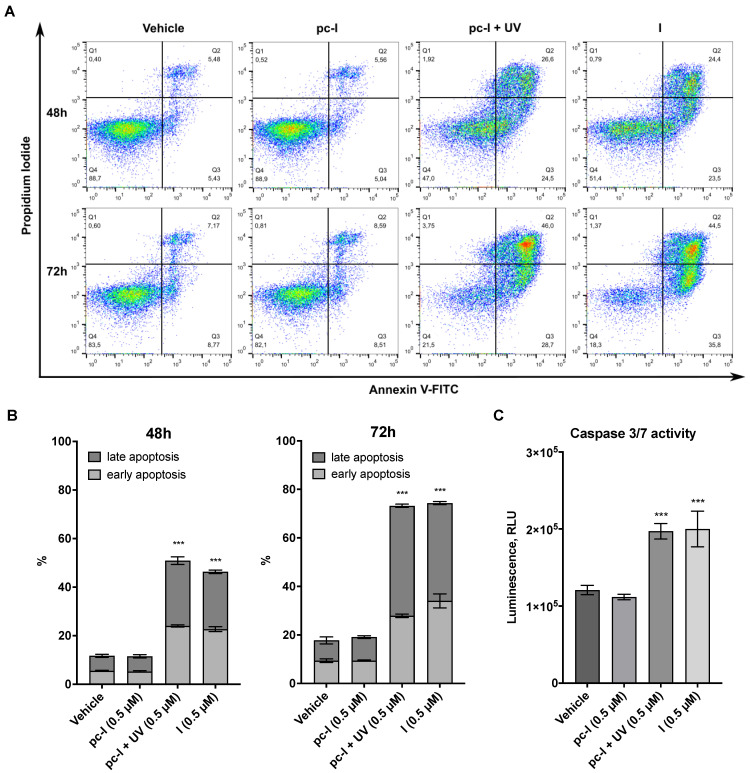
Results from apoptosis assays. A2780 cells were treated with 0.5 µM **pc-I**, **pc-I** + UV, **I** or vehicle (DMSO). (**A**) Cells were stained with annexin V and propidium iodide (PI) after 48 and 72 h. (**B**) Annexin-V-positive but PI-negative cells were classified as early apoptotic; annexin-V- and PI-positive cells were classified as late apoptotic. Statistical difference was calculated for annexin-V-positive cells compared to vehicle. (**C**) Cells were treated with the compounds and vehicle (DMSO) for 24 h, followed by incubation with caspase-Glo 3/7 substrate, and subsequently, luminescence was measured. For primary data, see Supporting Information Figure S2. Statistical analysis was performed with one-way ANOVA (*** *p* < 0.001).

Encouraged by the promising antiproliferative activity of **pc-I** in the presence of light, we investigated whether **I** and **pc-I** are capable of inducing apoptosis. To this end, A2780 cells were treated for 48 or 72 h with 0.5 µM of **I** and **pc-I**, stained with annexin V/propidium iodide (PI), and afterwards analyzed with flow cytometry. The results are presented in Figure 6A,B. Treatment with **I** led to a significant increase in early and late apoptotic cells compared to the DMSO control, while **pc-I** in the absence of light showed no significant increase in early and late apoptosis. However, after light activation, **pc-I** also demonstrated increased apoptosis levels which were comparable to compound **I**. 

Finally, we investigated whether the apoptosis induction of **I** and **pc-I** + UV is mediated in a caspase 3/7-dependent fashion by performing caspase-Glo 3/7 activation assays. The treatment of A2780 cells with **I** or **pc-I** + UV led to a significant activation of caspase 3/7, while **pc-I** was inactive in the absence of UV light (Figure 6C). Taken together, the results demonstrate that light-activated **pc-I** can kill cancer cells via apoptosis induction and in a caspase 3/7-dependent fashion. 

## 3. Discussion

In this work, we have designed and synthesized light-activatable prodrugs of our previously reported HDACi **DDK-137** (**I**) and **VK1** (**II**) by attaching a DMNB group to the zinc-binding moiety of both inhibitors. Through irradiation with light at 365 nm, **pc-I** showed fast deprotection and thereby conversion to its parent inhibitor **I,** while **pc-II** decomposed. In subsequent fluorogenic assays against human HDAC1 and HDAC6, **pc-I** showed only low inhibitory activity. After irradiation with light, the inhibitory activity of **pc-I** strongly increased against both isoforms (HDAC1: 121-fold; HDAC6: 37-fold), leading to IC_50_ values comparable to those observed for the parent inhibitor **I**. 

For subsequent evaluation of **I** and phototreated **pc-I**, we used the melanoma cell line MV-3 and the ovarian cancer cell line A2780. The melanoma cell line was chosen since this cancer entity is easily accessible with light, while the ovarian cancer cell line was selected because we previously showed that A2780 cells are highly sensitive to our peptoid-based HDACi [7]. Both **I** and photoactivated **pc-I** demonstrated pronounced inhibition of cellular HDAC activity in a whole-cell HDAC inhibition assay in the melanoma cell line MV-3 and displayed IC_50_ values in the double-digit nanomolar concentration range; meanwhile, **pc-I** was inactive without irradiation with UV light. Similar results were observed when we studied intracellular HDAC inhibition with immunoblot analysis in the MV-3 cell line. Again, only **I** and photoactivated **pc-I**, but not **pc-I** in the absence of light, were capable of inducing a hyperacetylation of acetyl-α-tubulin (a marker of HDAC6 inhibition) and acetyl-histone H3 (a marker of HDAC1-3 inhibition). While the prodrug **pc-I** was inactive in MTT viability assays (MV-3 and A2780 cell lines) and annexin V/PI apoptosis assays (A2780 cells), phototreated **pc-I** showed antiproliferative activity and apoptosis induction comparable to that of the parent compound **I**. Caspase 3/7-activation assays in A2780 cells confirmed that **I** and light-activated **pc-I** can kill cancer cells via apoptosis induction in a caspase 3/7-dependent fashion. 

Compared to previously published photocaged HDACi such as Zap-Pano and p-Fc-SAHA [32], which utilize the same DMNB caging group, our light-activatable HDACi **pc-I** features some important advantages. The treatment of Zap-Pano with light of the wavelength of 405 nm generated a significant amount of the corresponding amide Pano-NH_2_ as a side product. In our decaging experiments, we did not observe this amide side product, thus indicating that **I** might be more suitable for photocaging than panobinostat. While the DMNB-caged ferrocene-containing HDACi p-Fc-SAHA showed encouraging decaging efficiency and activity in cell-free assays, insufficient aqueous solubility prevented the analysis of p-Fc-SAHA in a cellular environment [31]. 

To conclude, the prodrug **pc-I** presented in this study is a promising tool for the further development of new light-activatable HDACi prodrugs.

## 4. Materials and Methods

### 4.1. General Information and Chemistry

Chemicals were obtained from *abcr* GmbH (Karlsruhe, Germany), *Acros Organics* (Geel, Belgien), *Carbolution Chemicals* (Sankt Ingberg, Germany), *Sigma-Aldrich* (Steinheim, Germany,) *TCI Chemicals* (Eschborn, Germany) or *VWR* (Langenfeld, Germany) and used without further purification. Technical-grade solvents were distilled prior to use. For all HPLC purposes, acetonitrile in HPLC-grade quality (HiPerSolv CHROMANORM, *VWR*, Langenfeld, Germany) was used. Water was purified with a PURELAB flex^®^ (*ELGA VEOLIA*, Celle, Germany). Thin-layer chromatography (TLC) was carried out on prefabricated plates (silica gel 60, F_254_, *Merck*). Components were visualized either by irradiation with ultraviolet light (254 nm or 366 nm) or by staining appropriately. Column chromatography was carried out on silica gel (60 Å, 40–60 μm, *Acros Organics*, Geel, Belgien). If no solvent is stated, an aqueous solution was prepared with demineralized water. Mixtures of two or more solvents are specified as “solvent A”/“solvent B”, 3/1, *v*/*v*; meaning that 100 mL of the respective mixture consists of 75 mL of “solvent A” and 25 mL of “solvent B”. The uncorrected melting points were determined using a *Büchi* (Essen, Germany) Melting Point M-560 apparatus. 

**Nuclear magnetic resonance spectroscopy (NMR):** Proton (^1^H) and carbon (^13^C) NMR spectra were recorded either on a Bruker AVANCE 500 MHz at a frequency of 500 MHz (^1^H) and 126 MHz (^13^C), a Bruker AVANCE III HD 600 MHz at a frequency of 600 MHz (^1^H) and 151 MHz (^13^C), or a Bruker AVANCE III HD 400 MHz at a frequency of 400 MHz (^1^H), 75 MHz (^13^C). The chemical shifts are given in parts per million (ppm). As solvents, deuterated chloroform (CDCl_3_), deuterated methanol (methanol-*d*_4_) and deuterated dimethyl sulfoxide (DMSO-*d*_6_) were used. The residual solvent signal (CDCl_3_: ^1^H NMR: 7.26 ppm, ^13^C NMR: 77.1 ppm; DMSO-*d*_6_: ^1^H NMR: 2.50 ppm, ^13^C NMR: 39.52 ppm; methanol-*d*_4_: ^1^H NMR: 3.31 ppm, 4.87 ppm, ^13^C NMR: 49.00 ppm) was used for calibration. The multiplicity of each signal is reported as singlet (s), doublet (d), triplet (t), quartet (q), multiplet (m) or combinations thereof. Multiplicities and coupling constants are reported as measured and might disagree with the expected values. 

**Mass spectrometry:** High-resolution electrospray-ionization mass spectra (HRMS-ESI) were acquired with Bruker Daltonik GmbH micrOTOF coupled to a an LC Packings Ultimate HPLC system and controlled by micrOTOFControl3.4 and HyStar 3.2-LC/MS, with a Bruker Daltonik GmbH ESI-qTOF Impact II coupled to a Dionex UltiMateTM 3000 UHPLC system and controlled by micrOTOFControl 4.0 and HyStar 3.2-LC/MS or with a micrOTOF-Q mass spectrometer (*Bruker*, Bremen, Germany) with ESI-source coupled with an HPLC Dionex UltiMate 3000 (*Thermo Scientific*, Heysham, United Kingdom). Low-resolution electrospray-ionization mass spectra (LRMS-ESI) were acquired with an *Advion* expression^®^ compact mass spectrometer (CMS) coupled with an automated TLC plate reader Plate Express^®^ (*Advion*, Ithaca, NY, USA). 

**High performance liquid chromatography (HPLC)**: A *Thermo Fisher Scientific* (Heysham, United Kingdom) UltiMate^TM^ 3000 UHPLC system with a Nucleodur 100-5 C18 (250 × 4.6 mm, *Macherey Nagel*, Düren, Germany) with a flow rate of 1 mL/min and a temperature of 25 °C, or a 100-5 C18 (100 × 3 mm, Macherey Nagel, Düren, Germany) with a flow rate of 0.5 mL/min and a temperature of 25 °C with an appropriate gradient was used. For preparative purposes, an AZURA Prep. 500/1000 gradient system with a Nucleodur 110-5 C18 HTec (150 × 32 mm, *Macherey Nagel*, Düren, Germany) column with 20 mL/min was used. Detection was implemented with UV absorption measurement at wavelengths of λ = 220 nm and λ = 250 nm. Bidest. H_2_O (A) and MeCN (B) were used as eluents with an addition of 0.1% TFA for eluent A. **Purity:** The purity of all final compounds was 95% or higher. Purity was determined via HPLC with the Nucleodur 100-5 C18 (250 × 4.6 mm, *Macherey Nagel*, Düren, Germany) at 250 nm. After column equilibration for 5 min, a linear gradient from 5% A to 95% B in 7 min, followed by an isocratic regime of 95% B for 10 min, was used. 

The following compounds were synthesized according to literature and used without further purification: Methyl-4-({*N*-[2-(benzylamino)-2-oxoethyl]-4-(dimethylamino)-benzamido}-methyl)-benzoate (**5**), 4-({*N*-[2-(benzylamino)-2-oxoethyl]-4-(dimethylamino)-benzamido}-methyl)-benzoic acid (**6**), *N*-[2-(benzylamino)-2-oxoethyl]-4-(dimethylamino)-*N*-[4-(hydroxycarbamoyl)-benzyl]-benzamide (**DDK-137** (**I**)) [8], *N*-{4-[(2-aminophenyl)-carbamoyl]-benzyl}-*N*-[2-(benzylamino)-2-oxoethyl]-4-(dimethylamino)-benzamide (**VK1** (**II**)) [7].

### 4.2. Experimental Protocols

#### 4.2.1. (4,5-Dimethoxy-2-nitrophenyl)-methanol (**2**)

6-Nitroveratraldehyde (0.845 g, 4.00 mmol, 1.00 eq.) was dissolved in CH_2_Cl_2_ (20 mL). NaBH_4_ (0.303 g, 8.00 mmol, 2.00 eq.) was added, and the solution was stirred for 30 min at room temperature. After completion of the reaction, 1 M HCl (20 mL) was added and stirred for further 10 min. The resulting layers were separated, and the inorganic layer was extracted with CH_2_Cl_2_ (3 × 20 mL). The combined organic layers were washed with sat. NaCl-solution (20 mL) and dried over Na_2_SO_4_. After removal of the solvent under reduced pressure, the alcohol **2** (0.835 g, 3.92 mmol, 98%) was isolated as a yellow solid. **R_f_** (cyclohexane/ethyl acetate 1/1, *v*/*v*): 0.28; **mp**.: 141–143 °C; **^1^H NMR** (600 MHz, CDCl_3_) δ = 7.70 (d, *J* = 1.5 Hz, 1H), 7.18 (d, *J* = 1.5 Hz, 1H), 4.96 (d, *J* = 1.5 Hz, 2H), 4.02–3.99 (m, 3H), 3.97–3.94 (m, 3H), 2.41 (s, 1H); **^13^C NMR** (151 MHz, CDCl_3_) δ = 154.0, 148.1, 139.9, 132.4, 111.2, 108.3, 63.0, 56.6, 56.5; **LRMS-ESI (*m/z*):** [M+Na]^+^ calcd for C_9_H_11_NO_5_: 236.0, found: 236.0.

#### 4.2.2. 2-[(4,5-Dimethoxy-2-nitrobenzyl)oxy]-isoindoline-1,3-dione (**4**)

Alcohol **2** (0.426 g, 2.00 mmol, 1.00 eq.), PPh_3_ (0.603 g, 2.30 mmol, 1.15 eq.) and *N*-hydroxyphthalimide (0.375 g, 2.30 mmol, 1.15 eq.) were dissolved in THF (13 mL) and cooled to 0 °C. Over 15 min, DIAD (0.451 mL, 0.465 g, 2.30 mmol, 1.15 eq., 5 equal portions every 3 min) was added dropwise. The reaction was allowed to warm to room temperature and stirred for 4 h. After completion of the reaction, the solvent was removed under reduced pressure and the residue dissolved in CH_2_Cl_2_ (20 mL). The organic layer was washed with sat. Na_2_CO_3_ (20 mL) solution. The inorganic layer was extracted with CH_2_Cl_2_ (3 × 20 mL); the combined organic layers were washed with sat. NaCl-solution (20 mL) and dried over Na_2_SO_4_. After removal of the solvent under reduced pressure, the crude product was purified with column chromatography (cyclohexane/ethyl acetate 2/1 to 1/3, *v*/*v*) yielding **4** (0.716 g, 2.00 mol, quant.) as a yellow solid. **R_f_** (cyclohexane/ ethyl acetate 2/1, *v*/*v*): 0.22; **mp.**: 104–106 °C; **^1^H NMR** (500 MHz, DMSO-*d_6_*) δ = 7.87–7.80 (m, 4H), 7.68 (s, 1H), 7.52 (s, 1H), 5.55 (s, 2H), 3.90 (s, 3H), 3.88 (s, 3H); **^13^C NMR** (126 MHz, DMSO-*d_6_*) δ = 163.0, 152.7, 148.3, 140.4, 134.7, 128.5, 124.9, 123.2, 112.9, 108.1, 75.5, 56.2, 56.1; **LRMS-ESI (*m/z*):** [M+H]^+^ calcd for C_17_H_14_N_2_O_7_: 359.1, found: 359.0.

#### 4.2.3. *N*-[2-(Benzylamino)-2-oxoethyl]-*N*-(4-{[(4,5-dimethoxy-2-nitrobenzyl)oxy]carbamoyl}benzyl)-4-(dimethylamino)benzamide (**pc-I**)

The synthesis was conducted in the dark as much as possible. Building block **4** (0.120 g, 0.335 mmol, 1.50 eq.) was dissolved in CH_2_Cl_2_ (2.2 mL). Hydrazine-monohydrate (50% in H_2_O, 0.102 mL, 53.2 mg, 1.67 mmol, 7.50 eq.) was added and the solution stirred for 4 h at room temperature. The precipitate was removed by filtration and washed with CH_2_Cl_2_ (3 × 5 mL). The solvent was evaporated under reduced pressure. The remaining yellow solid was re-dissolved in CH_2_Cl_2_ (0.37 mL) and used without further purification (hydroxylamine solution).

In a separated vessel, carboxylic acid **6** (99.3 mg, 0.223 mmol, 1.00 eq) was dissolved in CH_2_Cl_2_ (0.37 mL). It was cooled to 0 °C before EDC*HCl (85.5 mg, 0.446 mmol, 2.00 eq.) was added and stirred for 10 min at this temperature, followed by the addition of the previously prepared hydroxylamine solution. The reaction mixture was stirred for a further 10 min at 0 °C followed by additional 2 h at room temperature. The solvent was removed under reduced pressure. The remaining oily residue was purified via preparative HPLC. **pc-I** (27.7 mg, 35.6 µmol, 16%) was isolated as a yellow lyophilizate. **^1^H NMR** (600 MHz, DMSO-*d*_6_) δ = 11.80 (s, 1H), 8.42 (t, *J* = 6.0 Hz, 1H), 7.71 (d, *J* = 7.9 Hz, 2H), 7.69 (s, 1H), 7.49 (s, 1H), 7.38 (d, *J* = 7.9 Hz, 2H), 7.34–7.29 (m, 4H), 7.27–7.20 (m, 3H), 6.70–6.62 (m, 2H), 5.32 (s, 2H), 4.67 (s, 2H), 4.30 (d, *J* = 5.9 Hz, 2H), 3.93 (s, 3H), 3.87 (s, 3H), 2.93 (s, 6H); **^13^C NMR** (151 MHz, DMSO-*d_6_*) δ = 172.2, 168.6, 158.8, 158.6, 153.7, 151.6, 148.3, 141.9, 140.3, 139.7, 131.4, 129.0, 128.7, 127.7, 127.3, 111.9, 111.6, 108.5, 73.8, 56.8, 56.6, 42.6; **HRMS-ESI (*m/z*):** [M+H]^+^ calcd for C_35_H_37_N_5_O_8_: 656.2720, found: 656.2715; **HPLC**: t_R_ = 12.10 min (99.6% purity).

#### 4.2.4. 4,5-Dimethoxy-2-nitrobenzyl-{2-[4-({*N*-[2-(benzylamino)-2-oxoethyl]-4-(dimethylamino)benzamido}methyl)benzamido]phenyl}carbamate (**pc-II**)

The synthesis was conducted in the dark as much as possible. **VK-1** (52.3 mg, 97.6 µg, 1.00 eq.) and pyridine (8.65 µL, 8.49 mg, 0.107 mmol, 1.10 eq.) were dissolved in THF (1.2 mL) and cooled to 0 °C. NVOC-Cl (28.1 mg, 0.102 mmol, 1.05 eq.) was added, and the reaction mixture was stirred for 1 h at this temperature. The ice bath was removed, and the suspension was allowed to warm to room temperature and stirred for a further 18 h. The solvent was removed under reduced pressure and the residue dissolved in CH_2_Cl_2_ (10 mL). The solution was washed with sat. NaHCO_3_ (10 mL), 1 m HCl (10 mL), and sat. NaCl (10 mL) solution. The organic layer was dried over Na_2_SO_4_ and the solvent removed under reduced pressure. The residue was suspended in acetonitrile (10 mL) and the precipitate isolated by filtration and washed with acetonitrile (3 × 10 mL). **pc-II** (38.6 mg, 49.8 µmol, 51%) was isolated as a yellow solid. **mp.**: 159-161 °C, **^1^H NMR** (600 MHz, DMSO-*d*_6_) δ = 9.83 (s, 1H), 9.19 (s, 1H), 8.45 (d, *J* = 6.2 Hz, 1H), 7.95 (d, *J* = 7.9 Hz, 2H), 7.70 (s, 1H), 7.61–7.55 (m, 2H), 7.44 (d, *J* = 7.9 Hz, 2H), 7.36–7.33 (m, 3H), 7.32 (s, 1H), 7.28–7.22 (m, 4H), 7.21–7.18 (m, 2H), 6.66 (d, *J* = 8.9 Hz, 2H), 5.48 (s, 2H), 4.73 (s, 2H), 4.32 (d, *J* = 5.9 Hz, 2H), 3.93 (s, 2H), 3.85 (s, 3H), 3.79 (s, 3H), 2.93 (s, 6H); **^13^C NMR** (151 MHz, DMSO-*d*_6_) δ = 171.7, 168.1, 165.2, 153.7, 153.3, 151.2, 147.8, 141.5, 139.4, 139.2, 133.1, 131.5, 130.2, 128.6, 128.3, 128.0, 127.2, 127.2, 126.8, 126.1, 125.7, 124.6, 122.0, 110.9, 110.7, 108.2, 62.9, 56.1, 42.1; **HRMS-ESI (*m/z*):** [M+H]^+^ calcd for C_42_H_42_N_6_O_9_: 775.3092, found: 775.3086; **HPLC**: t_R_ = 12.75 min (97.2% purity).

#### 4.2.5. Benzyl (*S*)-{6-acetamido-1-[(4-methyl-2-oxo-2*H*-chromen-7-yl)amino]-1-oxohexan-2-yl}carbamate (**ZMAL**)

Cbz-l-Lys-OH (0.500 g, 1.75 mmol, 1.00 eq.) was dissolved in acetonitrile/water (27.5 mL 9/1, *v*/*v*). Acetic anhydride (0.200 mL, 0.214 g, 2.10 mmol, 1.20 eq.) was added and the solution stirred for 2 h at room temperature. The solution was diluted with water (125 mL), and the solvent was removed by lyophilization. The crude product and DIPEA (0.893 mL (0.663 g, 5.25 mmol, 3.00 eq.) were dissolved in DMF (4.4 mL). HATU (0.996 g, 2.62 mmol, 1.50 eq.) and 7-amino-4-methylcumarine (0.613 g, 3.50 mmol, 2.00 eq.) were added and the solution stirred for 16 h at room temperature. The solvent was removed under reduced pressure and the residue dissolved in ethyl acetate (50 mL). The organic layer was washed with 1 m HCl (2 × 30 mL), sat. NaCl (30 mL) solution, and dried over Na_2_SO_4_. The solvent was removed under reduced pressure and the residue purified by column chromatography (ethyl acetate/MeOH 1/0 to 93/7, *v*/*v*), yielding the target compound (0.537 g, 1.12 mmol, 64%) as an off-white solid. **R_f_** (ethyl acetate/ MeOH 95/5, *v*/*v*): 0.25; **^1^H NMR** (400 MHz, DMSO-d_6_) δ = 10.47 (s, 1H), 7.80 (t, J = 5.6 Hz, 1H), 7.77 (d, J = 2.0 Hz, 1H), 7.72 (d, J = 8.7 Hz, 1H,), 7.64 (d, J = 7.7 Hz, 1H), 7.49 (dd, J = 8.7, 2.1 Hz, 1H), 7.41–7.16 (m, 5H), 6.26 (d, J = 1.4 Hz,), 5.04 (s, 2H), 4.14 (td, J = 8.2, 5.0 Hz, 1H), 3.00 (td, J = 7.0, 3.4 Hz, 2H), 2.39 (d, J = 1.3 Hz, 3H), 1.76 (s, 3H), 1.73–1.56 (m, 2H), 1.45–1.23 (m, 4H). **^13^C-NMR** (75 MHz, DMSO-d_6_) δ = 179.2, 172.0, 169.0, 160.1, 156.2, 153.7, 153.1, 142.2, 128.4, 127.9, 127.8, 125.6, 115.8, 115.1, 112.3, 105.7, 65.5, 55.6, 38.3, 31.3, 28.9, 23.1, 22.6, 18.0; **HRMS-ESI (*m*/*z*):** [M+Na]^+^ calcd for C_26_H_29_N_3_O_6_: 502.1954, found: 502.1962; **HPLC**: t_R_ = 12.85 min (98.9% purity (300 nm)). Reported data match the literature [34].

### 4.3. Photolysis of Photocaged HDAC Inhibitor Prodrugs

Photolysis (photo-uncaging) of photocaged HDACi prodrugs was performed at room temperature using a UVP CL-1000 Crosslinker (Analytik Jena GmbH, Jena, Germany) equipped with five UV-A tubes (F8T5/BL368; λ_max_ = 365 nm; Analytik Jena GmbH, Jena, Germany). Hereafter, this entire experimental set-up will be referred to as the “UV reactor”.

#### Testing of Photo-Uncaging Efficiency

For the photo-uncaging efficiency testing, 10 µL of a 1.0 mM DMSO solution of the respective compound was irradiated for 10 min using the UV reactor described above. Subsequently, 90 µL acetonitrile was added, and the samples were analyzed via HPLC. The amount of photo-cleaved HDACi was determined by comparison with a five-point calibration curve of the respective parent inhibitor, using HPLC/UV detection at an appropriate wavelength (area under the curve; area mAu*min).

### 4.4. Biological Evaluation

#### 4.4.1. HDAC Enzyme Inhibition Assays

First, predilutions (100-fold final assay concentration) of test compounds or reference inhibitors were prepared. To this end, 3-fold serial dilutions of the respective DMSO stock solutions were prepared in DMSO using clear V-bottom 96-well microplates (*Greiner Bio-One*, Greiner AG, Kremsmünster, Austria). Predilutions designated for photolysis were irradiated for 10 min using the UV reactor described above. Subsequently, all DMSO predilutions were further diluted 1:10 in assay buffer (50 mM Tris-HCl, pH 8.0, 137 mM NaCl, 2.7 mM KCl, 1 mM MgCl_2_, 0.1 mg/mL BSA). Next, 5.0 µL of these serial dilutions in assay buffer were transferred into 96-well microplates (OptiPlate-96 Black, PerkinElmer, Waltham, MA, USA). Then, 35 µL of the fluorogenic substrate ZMAL (Z-Lys(Ac)-AMC (see Section 4.2.5); 21.43 μM in assay buffer) and 10 μL of human recombinant HDAC1 (BPS Bioscience Inc., San Diego, CA, USA, Catalog# 50051) or human recombinant HDAC6 (*BPS Bioscience* Inc., San Diego, CA, USA, Catalog# 50006) enzyme solution in assay buffer were added. The total assay volume (50 µL, 1% DMSO) was incubated at 37 °C for 90 min. Subsequently, 50 μL of trypsin (0.4 mg/mL) in trypsin buffer (50 mM Tris-HCl, pH 8.0, 100 mM NaCl) was added, followed by additional 30 min of incubation at 37 °C. Fluorescence (excitation: 355 nm, emission: 460 nm) was measured using a FLUOstar OPTIMA microplate reader (*BMG LABTECH* GmbH, Ortenberg, Germany). IC_50_ values were determined by generating normalized dose–response curves using the built-in *“log(inhibitor)* vs. *response (three parameters)”* equation provided by GraphPad Prism (GraphPad Prism 9.0, San Diego, CA, USA). All compounds were tested in duplicates; reported mean IC_50_ values, including standard deviation, were calculated from at least two independent experiments. 

#### 4.4.2. Cell Line and Cell Culture

The amelanotic melanoma cell line MV-3 was obtained from EPO GmbH Berlin-Buch. The human ovarian carcinoma cell line A2780 was obtained from the European Collection of Cell Cultures (*ECACC*, Salisbury, UK). The cell lines were cultivated in RPMI 1640 medium supplemented with 10% fetal bovine serum and 50 U/mL penicillin and 50 µg/mL streptomycin and incubated at 37 °C under humidified air with 5% CO_2_ to 80–90% confluency before being used in further assays.

#### 4.4.3. HDAC Whole-Cell Assay

The HDAC whole-cell assay is based on the assay established by *Ciossek* et al. [35] and *Bonfils* et al. [36], with minor changes. Melanoma cells MV-3 were seeded in concentration of 15 × 10^3^ cells/well (total volume of 81 µL) in 96-well cell-culture microplates (Catalog# 655086, *Greiner Bio-One*, Frickenhausen, Germany ) and cultured for 24 h. Aliquots of the DMSO stock solutions (10 mM) designated for photolysis were irradiated for 10 min with the UV reactor (see Section 4.3). The stock solutions processed with or without irradiation were used to perform a 4-fold serial dilution in PBS. Next, the cells were treated with 9 µL of the serial dilution for 18 h. For the analysis of the remaining HDAC activity, 10 µL of substrate solution, containing 3 mM Boc-Lys(ε-Ac)-AMC (Catalog# 233691-67-3, *BLD pharmatech* GmbH, Kaiserslautern, Germany) and 0.5% IGEPAL CA-630 (Catalog# J61055, *Alfa Aesar*, *Thermo Fisher Scientific*, Heysham, United Kingdom), was added and incubated for 3 h under cell culture conditions. The reaction was stopped with 100 µL of stop solution (50 mM Tris-HCl, 137 mM NaCl, 2.7 mM KCl, 1 mM MgCl_2_, 1% IGEPAL CA-630, 10 µM vorinostat, 2.0 mg/mL trypsin) and developed for 1.5 h under cell culture conditions. The fluorescence signal was measured using a Spark multimode microplate reader (*Tecan* Group AG, Männedorf, Swiss) at excitation wavelength of λ = 355 nm and emission wavelength of λ = 460 nm. Concentration-effect curves were generated using the four-parameter logistic equation (GraphPad Prism 9.0, San Diego, CA, USA), and mean IC_50_ values were determined by nonlinear regression based on at least three independent experiments in duplicates.

#### 4.4.4. MTT Cell Viability Assay

For this, 200-fold dilution series of the compounds of the respective stock solutions in DMSO (10 mM) were prepared in DMSO using clear U-bottom 96-well microplates (*Greiner Bio-One*, Greiner AG, Kremsmünster, Austria). Predilutions intended for photolysis experiments were irradiated for 10 min in the UV reactor described above. Thereafter, all predilutions were further diluted 1:200 in medium and provided in clear sterile F-bottom 96-well plates (*Starlab GmbH*, Hamburg, Germany). MV-3 cells were added at a density of 3000 cells/well and A2780 cells at a density of 8000 cells/well. The cells were added directly and without prior attachment. Final volume was 200 µL, and the final DMSO concentration was 0,5%. After 72 h, 40 µL MTT (3-(4,5-dimethylthiazol-2-yl)-2,5-diphenyltetrazolium bromide; *BioChemica*, Applichem GmbH, Darmstadt, Germany ) solution (5 mg/mL in phosphate-buffered saline) was added to determine cell survival. The formazan dye was dissolved in DMSO (*Sigma-Aldrich*, Steinheim, Germany) after 1 h, and absorbance was measured at 570 nm and 690 nm using a Multiskan microplate photometer (*Thermo Fisher Scientific*, Waltham, MA, USA). Concentration-effect curves were generated using the four-parameter logistic equation (*GraphPad* Prism 9.0, San Diego, CA, USA), and mean IC_50_ values were determined by nonlinear regression based on at least three distinct experiments in duplicates.

#### 4.4.5. Immunoblot

MV-3 cells were seeded to cell culture flasks and cultured for 48 h. Aliquots of the DMSO stock solution (10 mM) designated for photolysis were irradiated for 10 min with the UV reactor (see Section 4.3). The cells were treated with the stock solutions, processed with or without irradiation, of the indicated concentration or vehicle (DMSO) for 24 h. Cell lysis was performed with Cell Extraction Buffer (10 mM Tris, pH 7.4, 100 mM NaCl, 1 mM EDTA, 1 mM EGTA, 1 mM NaF, 20 mM Na_4_P_2_O_7_, 2 mM Na_3_VO_4_, 1% Triton™ ×-100, 10% glycerol, 0.1% SDS, 0.5% deoxycholate; Catalog# FNN0011, *Thermo Fisher Scientific Inc*., Waltham, MA, USA) and addition of Halt Protease Inhibitor Cocktail (100×) (Catalog# 78429, *Life Technologies GmbH* Carlsbad, CA, USA) and phenylmethanesulfonyl fluoride (Catalog# 10837091001, *Sigma-Aldrich*, St. Louis, MO, USA) according to manufacturer instructions. Protein content was determined by Pierce™ BCA Protein Assay Kit (Catalog# 23225, *Thermo Fisher Scientific Inc*., Waltham, MA, USA) according to manufacturer guidelines. Samples were denatured by Laemmli 2× Concentrate (Catalog# S3401-10VL, *Sigma-Aldrich*, St. Louis, MO, USA), and Precision Plus Protein Unstained Standard was used as molecular weight marker (Catalog# 1610363, *Bio-Rad*, Hercules, CA, USA). SDS-PAGE was performed with 10% Mini-PROTEAN TGX Stain-Free Gel at 200 V for 50 min (Catalog# 458035, *Bio-Rad*, Hercules, CA, USA). Afterwards, proteins were transferred with the Trans-Blot Turbo Transfer System (Catalog# 1704150, *Bio-Rad*, Hercules, CA, USA) to Immobilon-FL PVDF membranes (Catalog# IPFL00005, *Millipore Merck*, Burlington, MA, USA) at 1.0 A for 30 min and incubated with 5% milk-powder solution for 1 h at room temperature under slight agitation. Subsequently, the membranes were incubated with anti-acetyl-histone H3 (Catalog# 9677S, *Cell Signaling Technology*, Denver, MA, USA), anti-acetyl-α-tubulin (Catalog#5335, *Cell Signaling Technology*, Denver, MA, USA), and anti-GAPDH (Catalog# T0004, *Affinity Biosciences*, Cincinnati, OH, USA) in 1:1000–1:10000 dilutions at 4 °C, overnight. Incubation with HRP-conjugated secondary anti-mouse (Catalog# sc-516102, *Santa Cruz*, Dallas, TX, USA) and anti-rabbit (Catalog# HAF008, *R&D Systems, Inc.*, Minneapolis, MN, USA) was performed for 1.5 h, and membranes were developed with clarity western ECL substrate (Catalog# 1705061, *Bio-Rad*, Hercules, CA, USA). ChemiDoc XRS+ System (Catalog# 1708265, *Bio-Rad*, Hercules, CA, USA) was used for detection and Image Lab Software 6.1 (*Bio-Rad*, Hercules, CA, USA) for analyses. 

#### 4.4.6. Annexin V/PI Assay

A2780 cells (4 × 10^4^ cells/mL) were seeded in 6-well plates (*Starlab GmbH*, Hamburg, Germany) and allowed to attach. First, dilutions (500 µM) in DMSO were prepared from the respective stock solutions in DMSO (10 mM). Predilutions for photolysis experiments were irradiated in clear U-bottom 96-well microplates (*Greiner Bio-One*, Greiner AG, Kremsmünster, Austria) for 10 min in the UV reactor described above. Subsequently, vehicle control (DMSO) or compounds, at a concentration of 0.5 µM, were added to the cells and incubated for 48 h and 72 h, respectively. The final volume was 3 mL, and the final DMSO concentration was 0.1%. Staining was performed in triplicate in two separate experiments using annexin V and propidium iodide and analyzed by flow cytometry (*Guava*^®^ easyCyte^TM^, Luminex, Austin, TX, USA), according to the manufacturer’s protocol (*BioLegend*, San Diego, CA, USA). 

Results were expressed as mean with standard deviation (SD). Statistical analysis was performed using GraphPad Prism (*GraphPad* Software, San Diego, CA, USA). Normality of the data was analyzed using the Shapiro–Wilk’s test. Statistical significance was assessed using one-way analysis of variance (ANOVA) with Tukey’s multiple comparison test. Differences were considered statistically significant at * *p* < 0.05, ** *p* < 0.01, *** *p* < 0.001.

#### 4.4.7. Caspase-Glo 3/7 Assay

A2780 cells (8000 cells/well) were seeded in white sterile 96-well plates (*ThermoFisher Scientific*, Waltham, MA, USA, #136102). Dilutions (500 µM) in DMSO were prepared from the respective stock solutions in DMSO (10 mM). Predilutions used for photolysis experiments were irradiated in clear U-bottom 96-well microplates (*Greiner Bio-One*, Greiner AG, Kremsmünster, Austria) for 10 min in the UV reactor described above. Vehicle control (DMSO) or compounds, at a concentration of 0.5 µM, were added to the cells. The cells were then incubated for 24 h under standard culture conditions. The final volume was 100 µL, and the final DMSO concentration was 0.1%. After 24 h, cells were subsequently incubated with Caspase-Glo 3/7 substrate (*Promega*, Madison, WI, USA, #G8091) for 1 h at 25 °C, according to the manufacturer´s protocol. Luminescence was detected at 25 °C using a Spark microplate reader (*Tecan* Group AG, Maennedorf, Swiss). Two independent experiments were performed in triplicates. 

GraphPad Prism (*GraphPad* Software, San Diego, CA, USA) was used to perform statistical analysis. Normality of the data was assessed using Shapiro–Wilk’s and Kolmogorov–Smirnov tests. To determine statistical differences between means, a one-way analysis of variance (ANOVA) was performed. If the difference was significant (*p* < 0.05), the mean of each column was compared to the mean of the control and corrected for multiple comparisons using Dunnett’s post-hoc test. Differences were considered statistically significant at * *p* < 0.05, ** *p* < 0.01, *** *p* < 0.001.

## Figures and Tables

**Table 1:** Inhibitory activity of photocaged **pc-I** (with and without UV irradiation) and **I** against human HDAC1 and HDAC6 *^a^*.

	pc-I	pc-I + UV	I	Vorinostat
IC_50_ (HDAC1) [nM]	824 ± 32.2	6.8 ± 1.2	12.7 ± 0.7	104 ± 6.2
IC_50_ (HDAC6) [nM]	794 ± 74.7	21.6 ± 2.8	13.1 ± 0.9	36.3 ± 2.8

*^a^* Reported mean IC_50_ values, including standard deviation, are calculated from at least two independent experiments. For primary data see Supporting Information Figure S1.

**Table 2:** Inhibitory activity of photocaged **pc-I** (with and without UV irradiation) and **I** against the MV-3 and A2780 cell line *^a^*.

	pc-I	pc-I + UV	I
IC_50_ (MV-3) [nM]	n.e. ^b^	727 ± 133	616 ± 161
IC_50_ (A2780) [nM]	n.e. ^b^	428 ± 62.5	249 ± 84.7

*^a^* Stated IC_50_ values were determined from the mean of three independent experiments in duplicates. UV irradiation was executed at 365 nm for 10 min. ^b^ n.e— no effect: <25% inhibition at 25 µM.

## Data Availability

Data is contained within the article and Supplementary Material.

## Supplementary Materials

The following Supporting Information can be downloaded at https://www.mdpi.com/article/10.3390/ph16030356/s1: Figure S1. Normalized dose–response curves of the in vitro HDAC inhibition assays against HDAC1 and HDAC6; Figure S2. Caspase-Glo 3/7 assays; Figure S3. Original images of acetylated α-tubulin immunoblots. Figure S4. Original images of acetylated histone H3 immunoblots. Figure S5. Calibration curve of DDK-137 (I).; HPLC traces and ^1^H and ^13^C NMR spectra of final compounds.
