# Supplementary material for: Photocaged Histone Deacetylase Inhibitors as Prodrugs in Targeted Cancer Therapy
Source: Pharmaceuticals (Basel). 2023 Feb 25;16(3):356. doi: 10.3390/ph16030356 (PMC10056348; doi:10.3390/ph16030356)
Supplement: Supplementary file 1 [file pharmaceuticals-16-00356-s001.zip › pharmaceuticals-2203775-supplementary.pdf]

Electronic Supplementary Information

Photocaged histone deacetylase inhibitors as prodrugs in targeted cancer therapy

Fabian B. Kraft [1], Maria Hanl [1], Felix Feller [1], Linda Schäker-Hübner [1] and Finn K. Hansen [1,]*

[1] Pharmaceutical Institute, Department of Pharmaceutical and Cell Biological Chemistry, University of Bonn, An der Immenburg 4, D-53121 Bonn, Germany

*Correspondence: finn.hansen@uni-bonn.de

Table of Contents

1. **Supplemental Figures** S3

2. **Photouncaging Experiments** S6

3. **HPLC Chromatograms** S8

4. **^{1}H and ^{13}C NMR Spectra** S9

1. Supplemental Figures

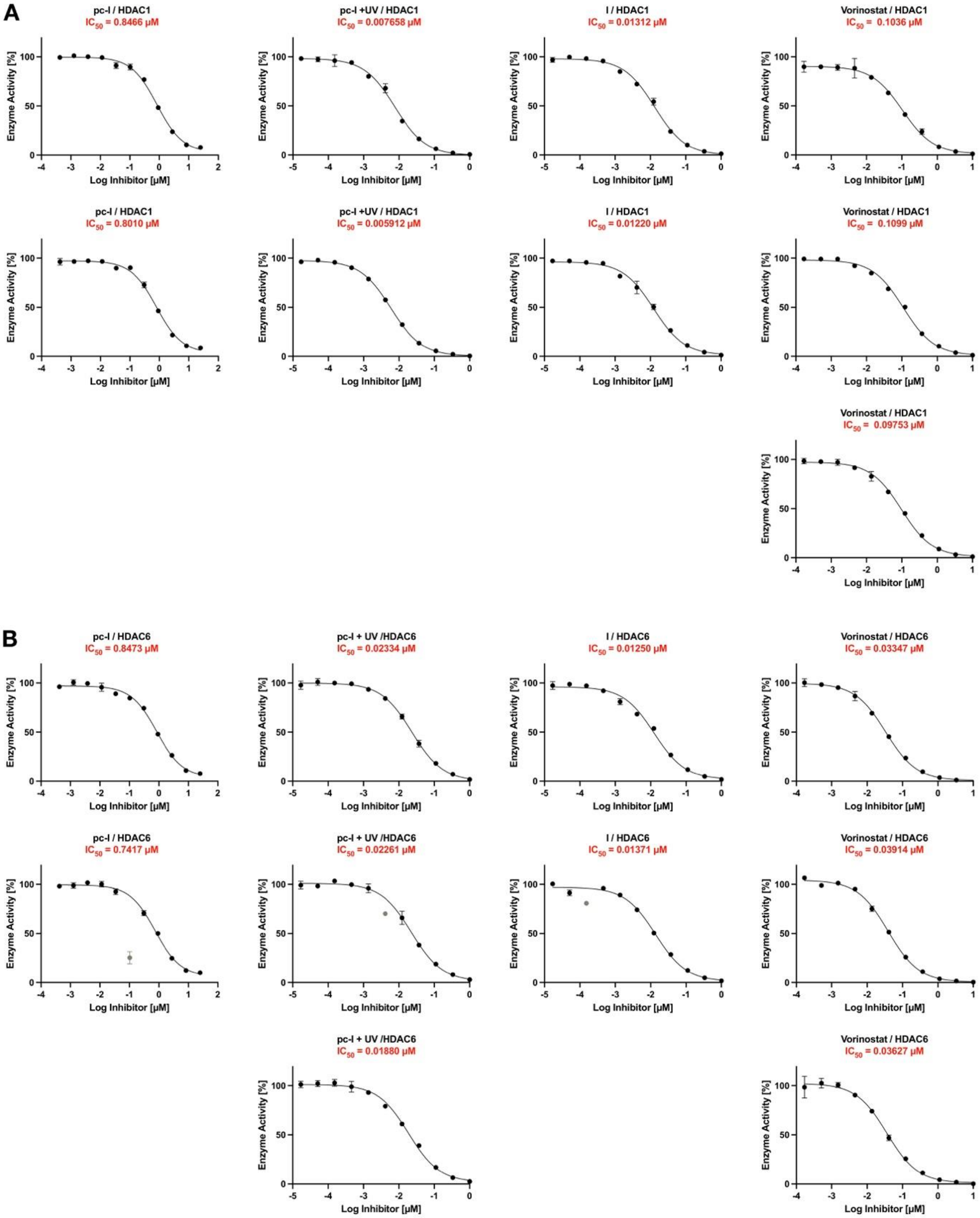

Figure S1. Normalized dose-response curves of the *in vitro* HDAC inhibition assays against HDAC1 (**A**) and HDAC6 (**B**). Data points which were used to calculate the IC_{50} values are depicted as black dots including the respective error bars (standard deviation). Excluded data points are depicted in grey. In some cases, more than two experiments were performed. Additionally, depending on the plate layout and the quantity of the investigated compounds, not all compounds were investigated in one assay run. Vorinostat was included in every assay run as a control compound.

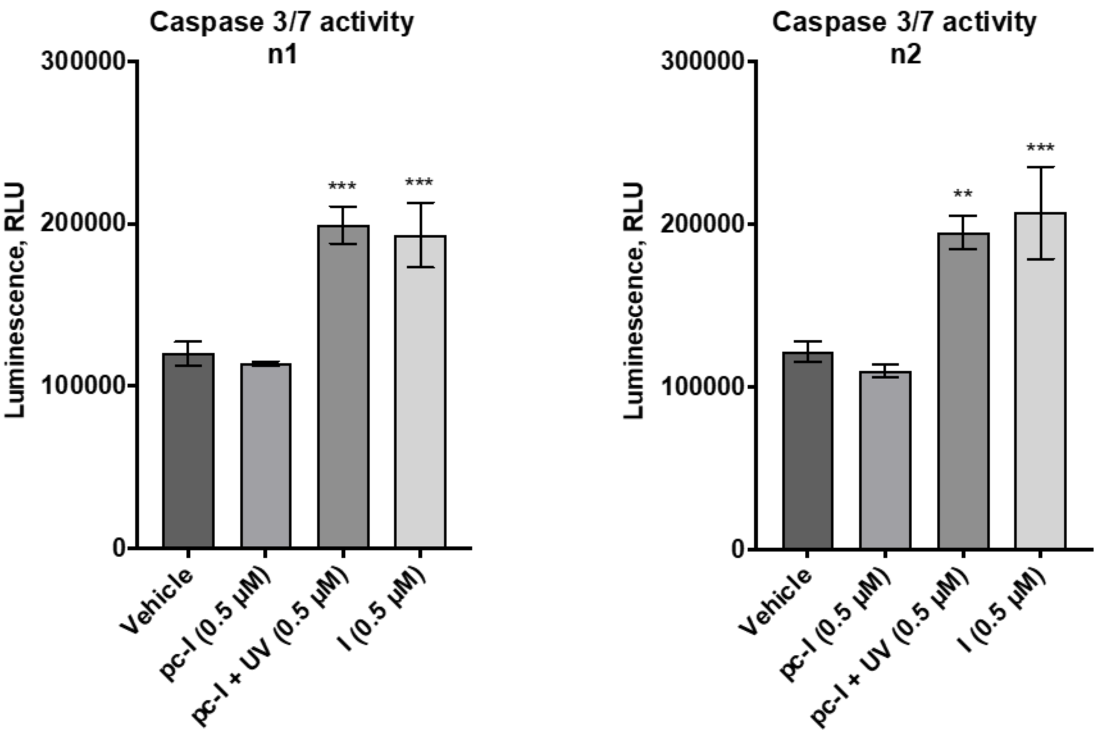

Figure S2. A2780 cells were treated with the indicated concentrations of pc-I, pc-I + UV, I and vehicle (DMSO) for 24h. Afterwards cells were incubated with Caspase-Glo 3/7 substrate and luminescence was determined. The assay was performed in two independent experiments (n1 and n2). Statistical analysis was calculated by one-way ANOVA (*p<0.05, **p<0.01, ***p<0.001).

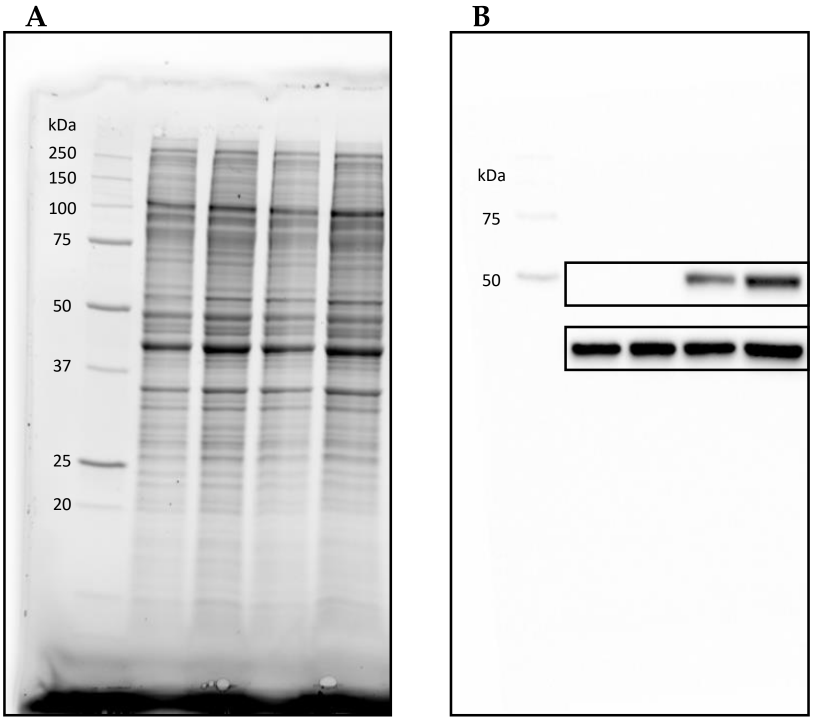

Figure S3. Original images of acetylated α-tubulin immunoblots. **A** shows the stain free detection image of the gel. **B** shows the image of the visualization of the selected acetylated α-tubulin and GAPDH bands.

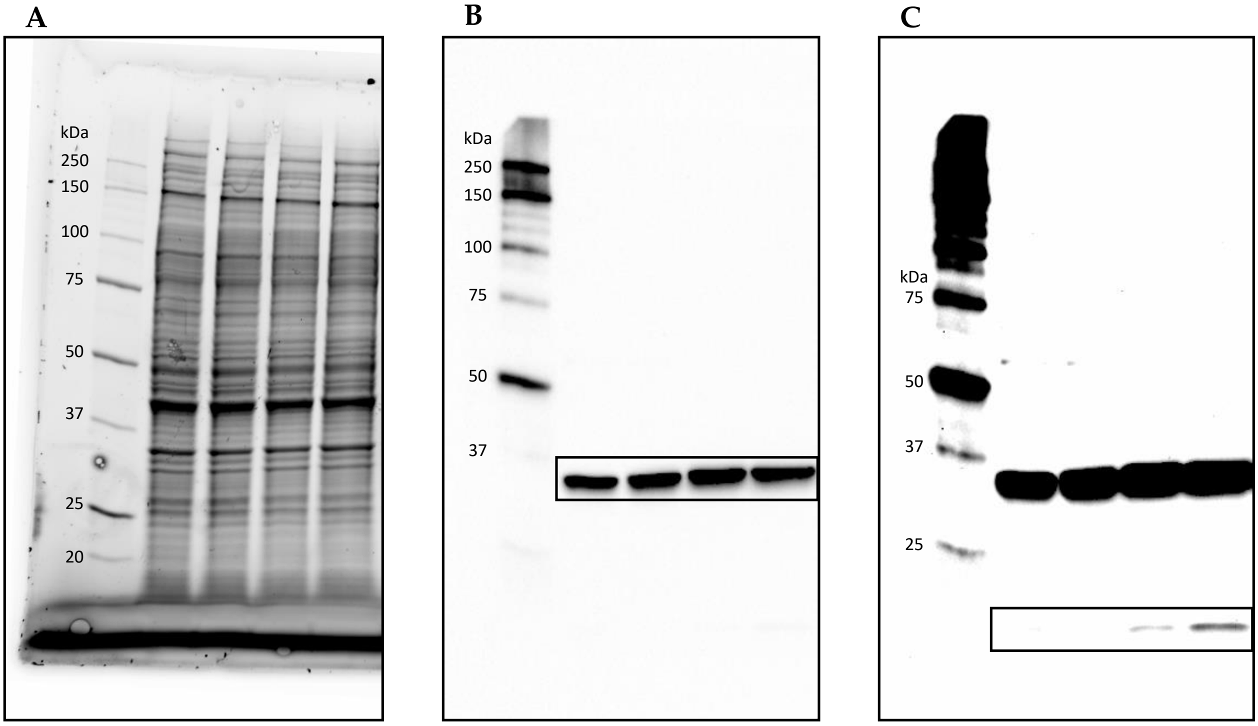

Figure S4. Original images of acetylated histone H3 immunoblots. **A** shows the stain free detection image of the gel. **B** shows the image of the visualization of the GAPDH band. **C** shows the image of the selected acetylated histone H3 band after longer visualization time.

2. Photouncaging Experiments

Photolysis (photouncaging) of photocaged HDACi prodrugs was performed at room temperature using an UVP CL-1000 Crosslinker (Analytik Jena GmbH, Jena, Germany) equipped with five UV-A tubes (F8T5/BL368; λmax = 365 nm; Analytik Jena GmbH, Jena, Germany). Hereafter, this entire experimental set-up will be referred to as the "UV-reactor".
For the deprotection experiments 10 µL of a 1.0 mM DMSO-solution of the respective compound were irradiated for 10 min using the UV-reactor described above. Subsequently, 90 µL acetonitrile were added and the samples were analyzed via HPLC. The amount of photo-cleaved HDACi was determined by comparison with a five-point calibration curve of the respective parent inhibitor using HPLC/UV detection at an appropriate wavelength (area under the curve; area mAu*min).

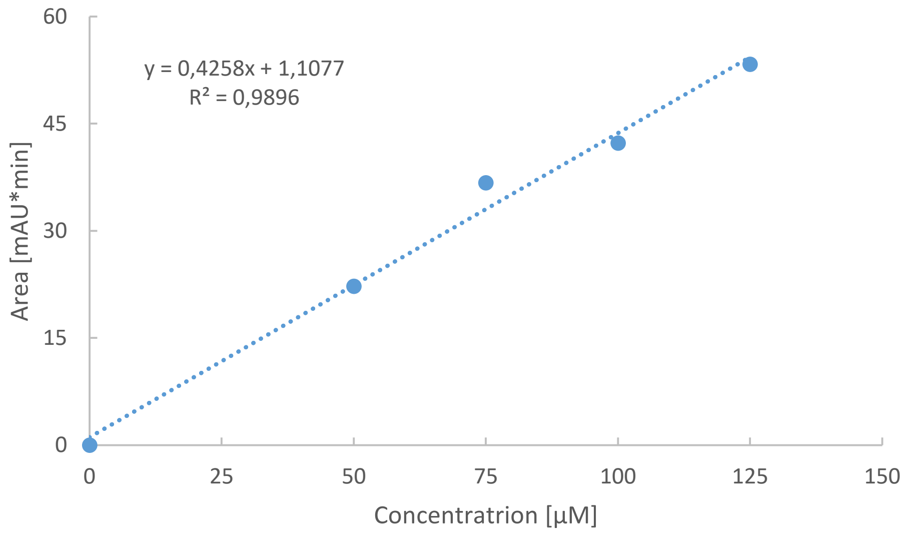

Figure S5. Calibration curve of **DDK-137** (**I**).

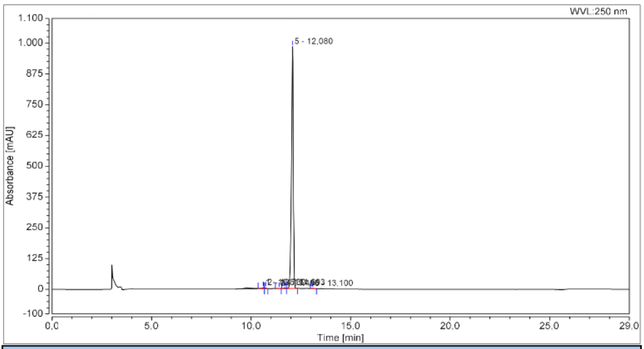

Integration Results

No.	Peak Name	Retention Time min	Area mAU*min	Height mAU	Relative Area %	Relative Height %	Amount n.a.
1		10,613	0,779	5,002	0,73	0,50	n.a.
2		10,730	0,315	3,802	0,29	0,38	n.a.
3		11,403	0,106	0,861	0,10	0,09	n.a.
4		11,693	0,359	3,049	0,33	0,31	n.a.
5		12,080	105,555	982,358	98,39	98,59	n.a.
6		13,100	0,167	1,382	0,16	0,14	n.a.
Total:			107,281	996,454	100,00	100,00	

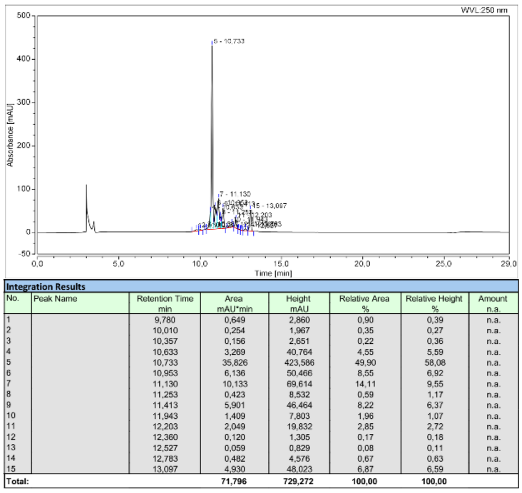

Integration Results

No.	Peak Name	Retention Time min	Area mAU*min	Height mAU	Relative Area %	Relative Height %	Amount n.a.
1		9,780	0,649	2,860	0,90	0,39	n.a.
2		10,010	0,254	1,967	0,35	0,27	n.a.
3		10,357	0,156	2,651	0,22	0,36	n.a.
4		10,633	3,269	40,764	4,55	5,59	n.a.
5		10,733	35,826	423,586	49,90	58,08	n.a.
6		10,953	6,136	50,466	8,55	6,92	n.a.
7		11,130	10,133	69,614	14,11	9,55	n.a.
8		11,253	0,423	8,532	0,59	1,17	n.a.
9		11,413	5,901	46,464	8,22	6,37	n.a.
10		11,943	1,409	7,803	1,96	1,07	n.a.
11		12,203	2,049	19,832	2,85	2,72	n.a.
12		12,360	0,120	1,305	0,17	0,18	n.a.
13		12,527	0,059	0,829	0,08	0,11	n.a.
14		12,783	0,482	4,576	0,67	0,63	n.a.
15		13,097	4,930	48,023	6,87	6,59	n.a.
Total:			71,796	729,272	100,00	100,00	

Figure S5. Representative chromatograms of **pc-I** before irradiation (left) and after 10 min of irradiation (right) with light at 365 nm. Initial concentration of **pc-I**: 100 μM. Concentration of photo-released **I** (t_R = 10.73 min) after irradiation: 81.5 μM (82%).

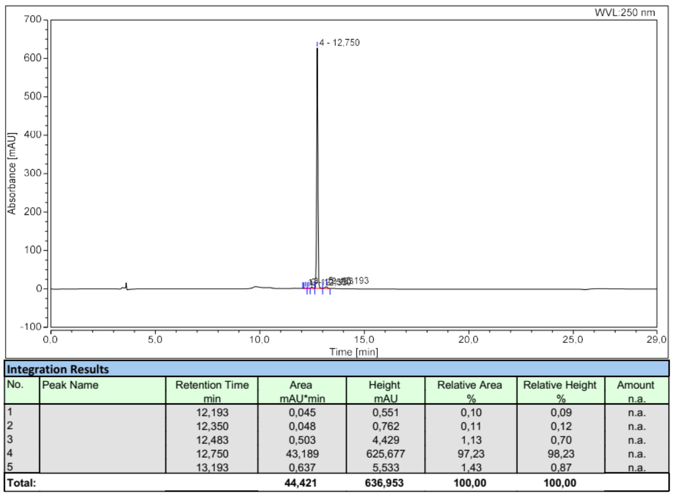

Integration Results

No.	Peak Name	Retention Time min	Area mAU*min	Height mAU	Relative Area %	Relative Height %	Amount n.a.
1		12,193	0,045	0,551	0,10	0,09	n.a.
2		12,350	0,048	0,762	0,11	0,12	n.a.
3		12,483	0,503	4,429	1,13	0,70	n.a.
4		12,750	43,189	625,677	97,23	98,23	n.a.
5		13,193	0,637	5,533	1,43	0,87	n.a.
Total:			44,421	636,953	100,00	100,00	

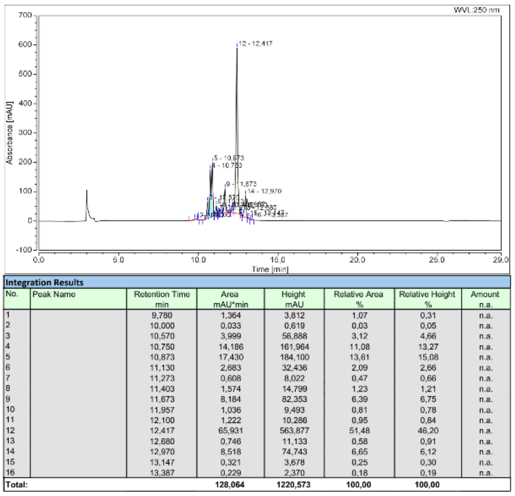

Integration Results

No.	Peak Name	Retention Time min	Area mAU*min	Height mAU	Relative Area %	Relative Height %	Amount n.a.
1		9,780	1,364	3,812	1,07	0,31	n.a.
2		10,000	0,033	0,619	0,03	0,05	n.a.
3		10,570	3,999	56,888	3,12	4,66	n.a.
4		10,750	14,186	161,964	11,08	13,27	n.a.
5		10,873	17,430	184,100	13,61	15,08	n.a.
6		11,130	2,683	32,436	2,09	2,66	n.a.
7		11,273	0,608	8,022	0,47	0,66	n.a.
8		11,403	1,574	14,799	1,23	1,21	n.a.
9		11,673	8,184	82,353	6,39	6,75	n.a.
10		11,957	1,036	9,493	0,81	0,78	n.a.
11		12,100	1,222	10,286	0,95	0,84	n.a.
12		12,417	65,931	563,877	51,48	46,20	n.a.
13		12,680	0,746	11,133	0,58	0,91	n.a.
14		12,970	8,518	74,743	6,65	6,12	n.a.
15		13,147	0,321	3,678	0,25	0,30	n.a.
16		13,387	0,229	2,370	0,18	0,19	n.a.
Total:			128,064	1220,573	100,00	100,00	

Figure S6. Representative chromatograms of **pc-II** before irradiation (left) and after 10 min of irradiation (right) with light at 365 nm. Initial concentration of **pc-II**: 100 μM. Concentration of photo-released **II** (t_R = 10.89 min) after irradiation: not detected.

3. HPLC Chromatograms

HPLC chromatogram of **pc-I** (purity: 99.6 %)

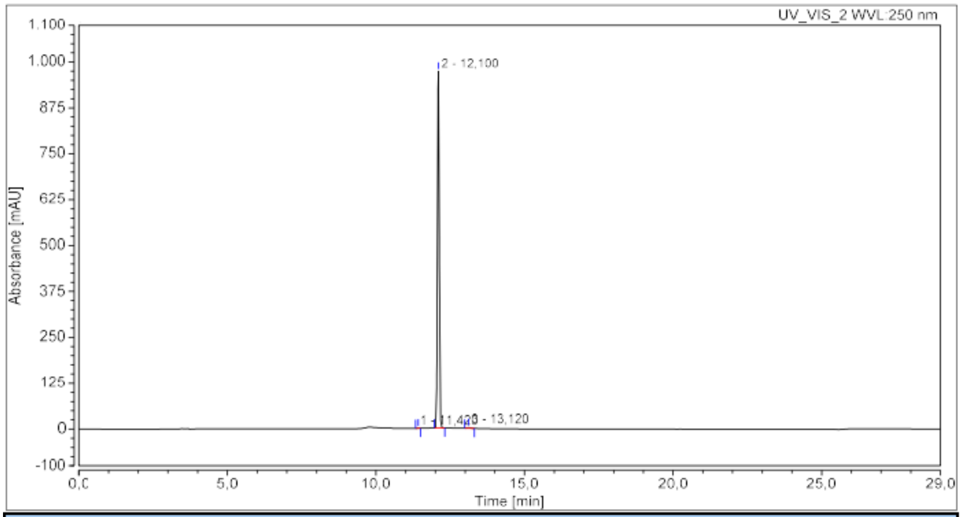

Integration Results

No.	Peak Name	Retention Time min	Area mAU*min	Height mAU	Relative Area %	Relative Height %	Amount n.a.
1		11,420	0,052	0,756	0,08	0,08	n.a.
2		12,100	66,624	971,696	99,57	99,69	n.a.
3		13,120	0,233	2,249	0,35	0,23	n.a.
**Total:**			**66,909**	**974,701**	**100,00**	**100,00**	

HPLC chromatogram of **pc-II** (purity: 97.2 %).

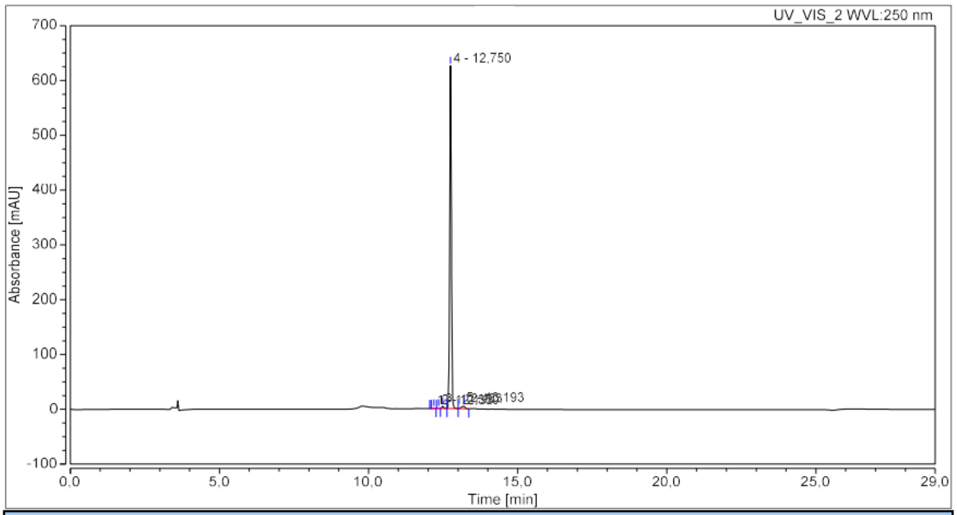

Integration Results

No.	Peak Name	Retention Time min	Area mAU*min	Height mAU	Relative Area %	Relative Height %	Amount n.a.
1		12,193	0,045	0,551	0,10	0,09	n.a.
2		12,350	0,048	0,762	0,11	0,12	n.a.
3		12,483	0,503	4,429	1,13	0,70	n.a.
4		12,750	43,189	625,677	97,23	98,23	n.a.
5		13,193	0,637	5,533	1,43	0,87	n.a.
**Total:**			**44,421**	**636,953**	**100,00**	**100,00**	

4. ^{1}H and ^{13}C NMR Spectra

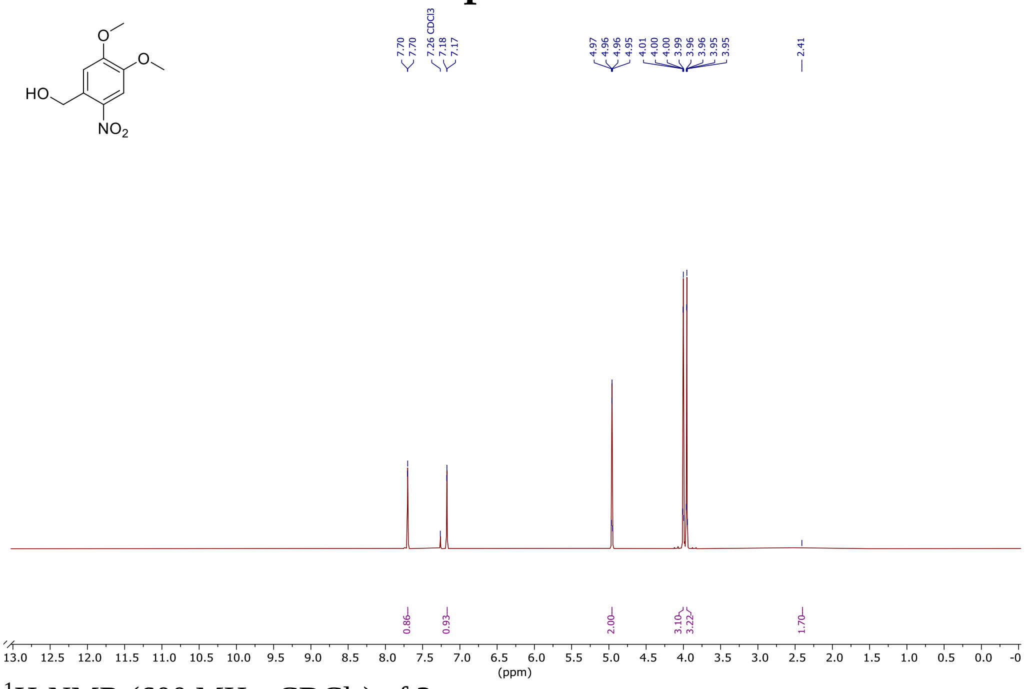

^{1}H-NMR (600 MHz, $CDCl_3$) of **2.**

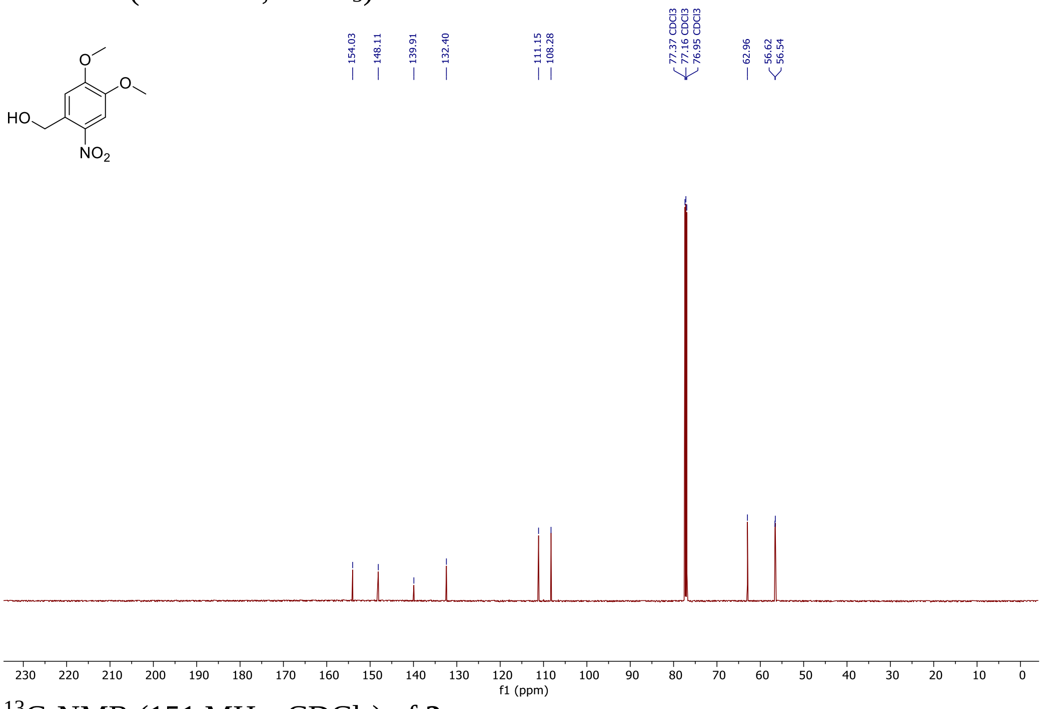

^{13}C-NMR (151 MHz, $CDCl_3$) of **2.**

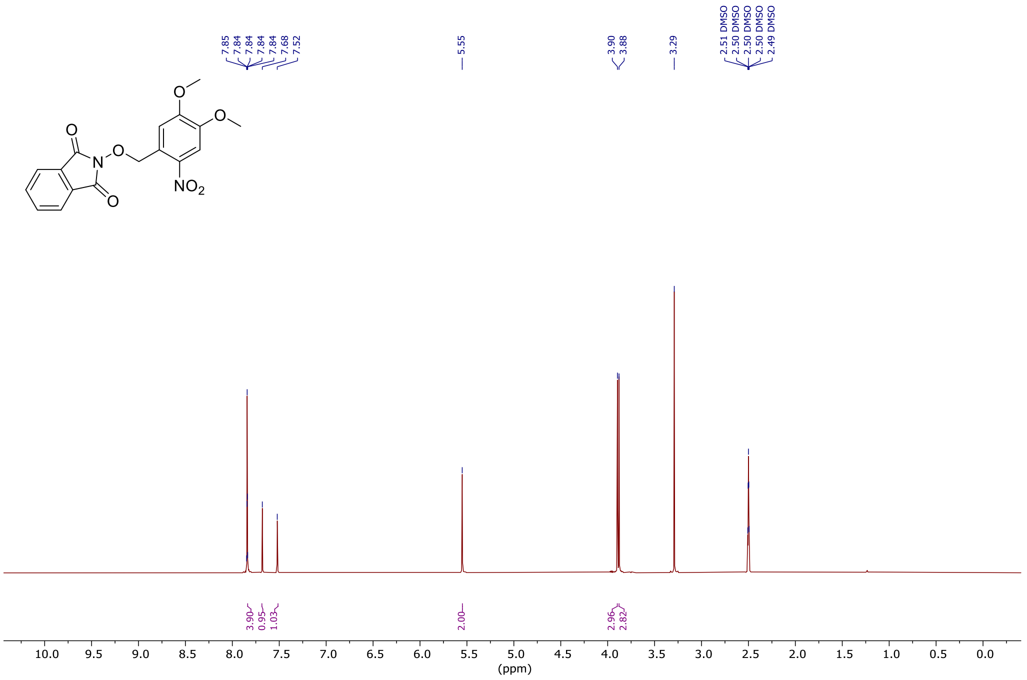

^{1}H-NMR (500 MHz, DMSO-d_6) of **4**.

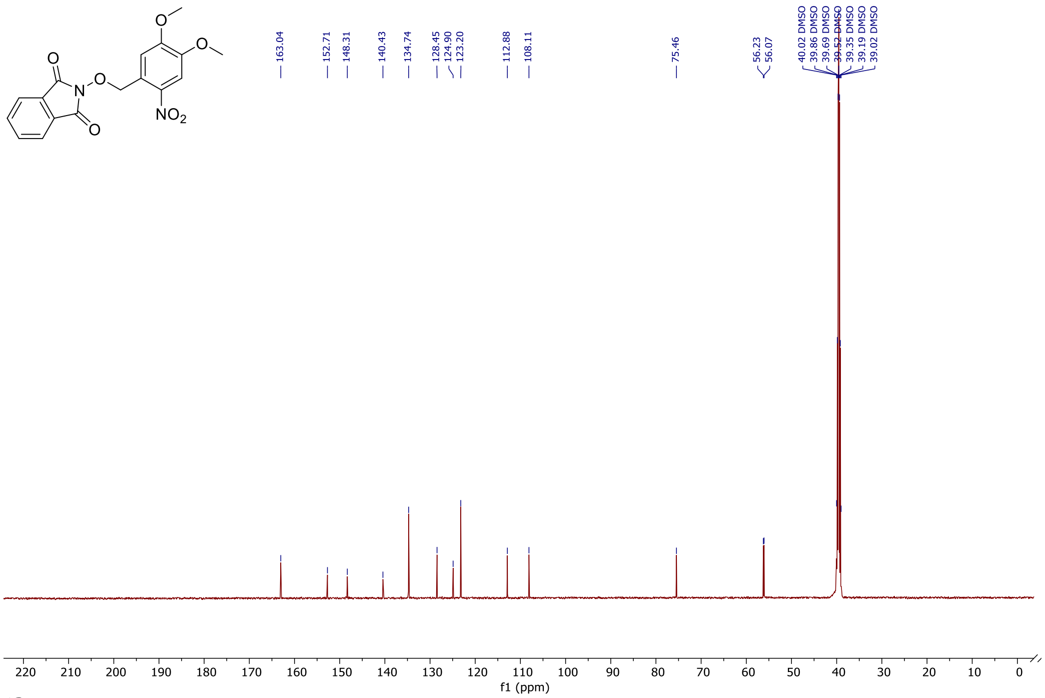

^{13}C-NMR (126 MHz, DMSO-d_6) of **4**.

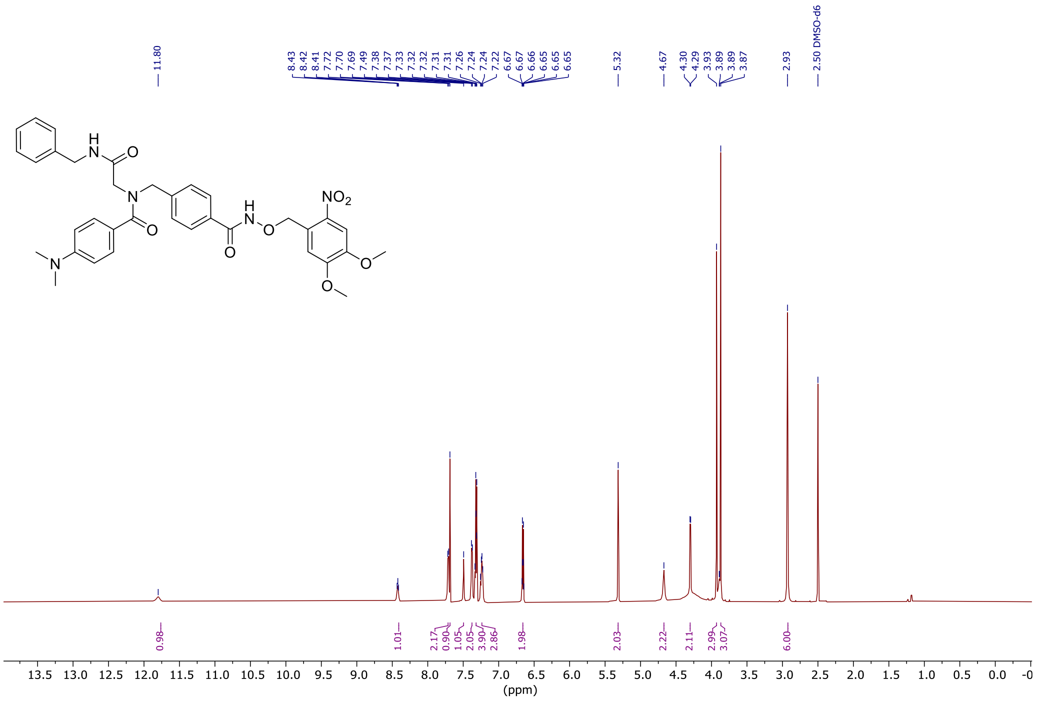

^{1}H-NMR (500 MHz, DMSO-d_6) of **pc-I.**

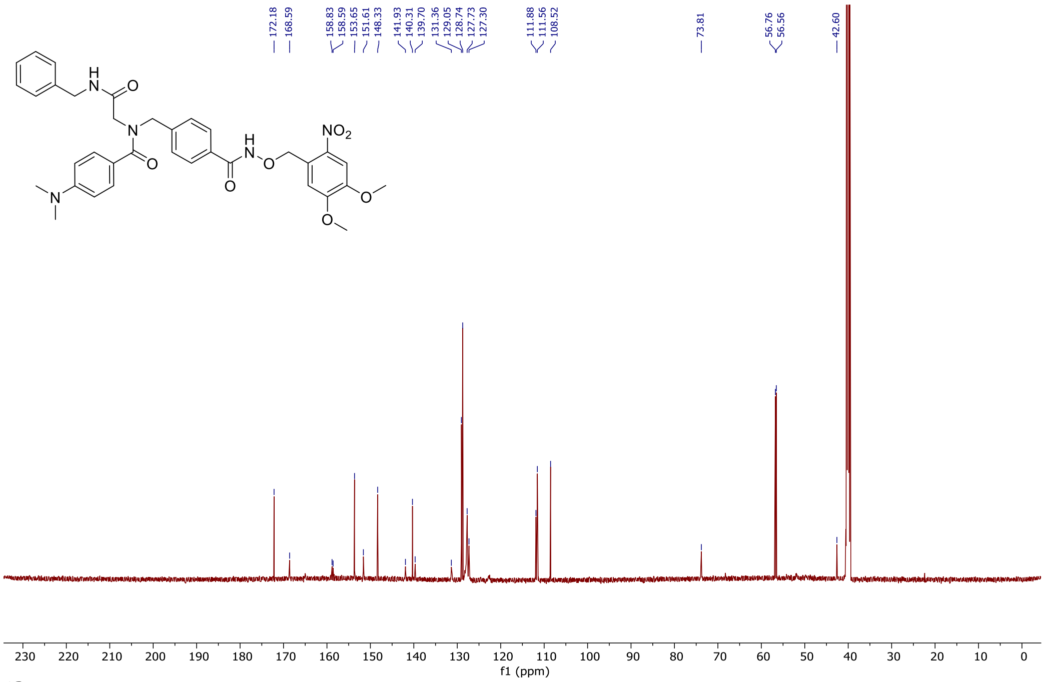

^{13}C-NMR (151 MHz, DMSO-d_6) of **pc-I.**

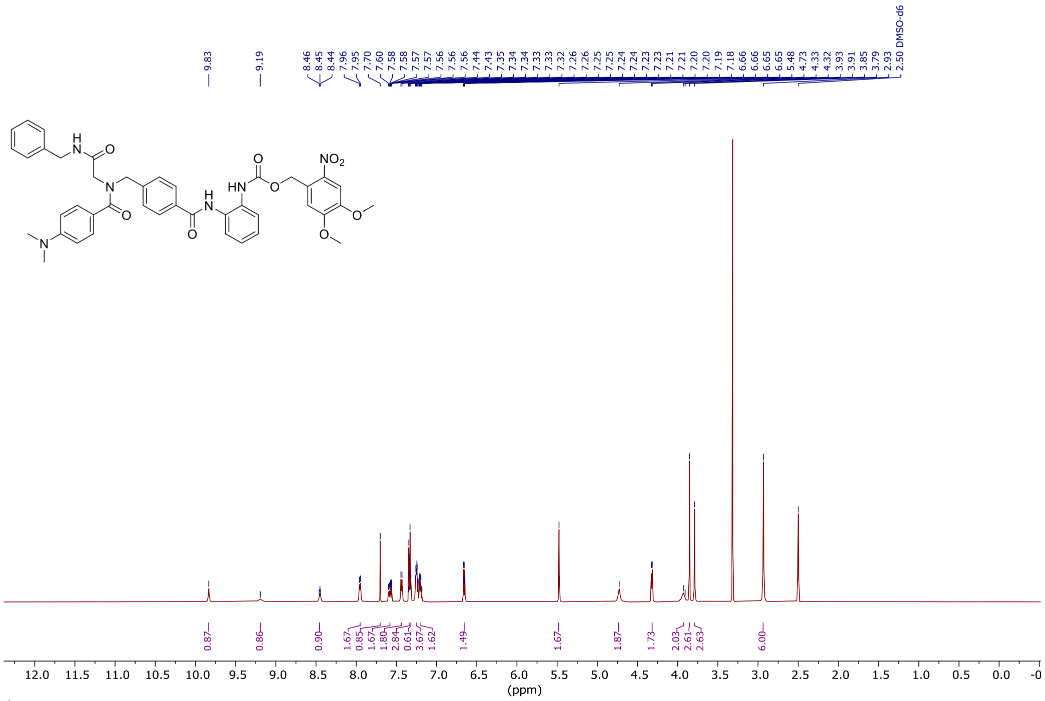

^{1}H-NMR (600 MHz, DMSO-d_6) of **pc-II**.

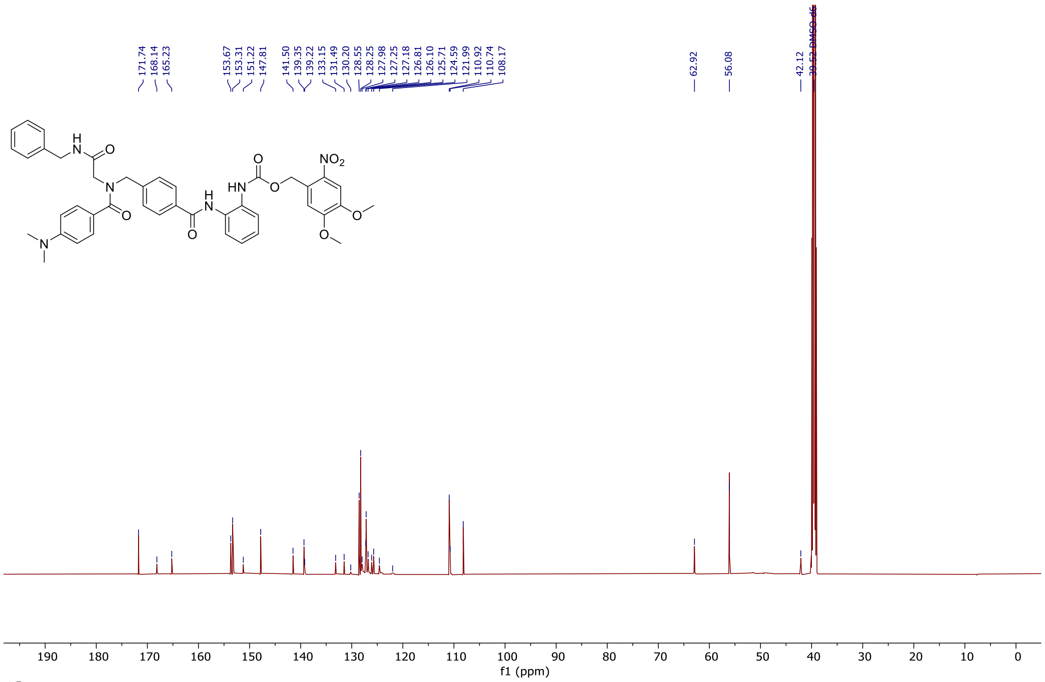

^{13}C-NMR (151 MHz, DMSO-d_6) of **pc-II**.
